# Pathogen-Induced Proapoptotic Phenotype and High CD95 (Fas) Expression Accompany a Suboptimal CD8^+^ T-Cell Response: Reversal by Adenoviral Vaccine

**DOI:** 10.1371/journal.ppat.1002699

**Published:** 2012-05-17

**Authors:** José Ronnie Vasconcelos, Oscar Bruña–Romero, Adriano F. Araújo, Mariana R. Dominguez, Jonatan Ersching, Bruna C. G. de Alencar, Alexandre V. Machado, Ricardo T. Gazzinelli, Karina R. Bortoluci, Gustavo P. Amarante-Mendes, Marcela F. Lopes, Mauricio M. Rodrigues

**Affiliations:** 1 Centro de Terapia Celular e Molecular (CTCMol), Universidade Federal de São Paulo-Escola Paulista de Medicina, São Paulo, São Paulo, Brazil; 2 Departmento de Microbiologia, Imunologia e Parasitologia, Universidade Federal de São Paulo-Escola Paulista de Medicina, São Paulo, São Paulo, Brazil; 3 Departamento de Microbiologia, Instituto de Ciências Biológicas, Universidade Federal de Minas Gerais, Belo Horizonte, Minas Gerais, Brazil; 4 Centro de Pesquisas René Rachou, FIOCRUZ, Belo Horizonte, Minas Gerais, Brazil; 5 Departamento de Bioquímica e Imunologia, Instituto de Ciências Biológicas, Universidade Federal de Minas Gerais, Pampulha, Belo Horizonte, Minas Gerais, Brazil; 6 Division of Infectious Disease and Immunology, Department of Medicine, University of Massachusetts Medical School, Worcester, Massachusetts, United States of America; 7 Departamento de Ciências Biológicas, Universidade Federal de São Paulo-Escola Paulista de Medicina, Diadema, São Paulo, Brazil; 8 Departamento de Imunologia, Instituto de Ciências Biomédicas, Universidade de São Paulo, São Paulo, São Paulo, Brazil; 9 Instituto de Biofísica Carlos Chagas Filho, Universidade Federal do Rio de Janeiro, Rio de Janeiro, Rio de Janeiro, Brazil; Washington University, United States of America

## Abstract

MHC class Ia-restricted CD8^+^ T cells are important mediators of the adaptive immune response against infections caused by intracellular microorganisms. Whereas antigen-specific effector CD8^+^ T cells can clear infection caused by intracellular pathogens, in some circumstances, the immune response is suboptimal and the microorganisms survive, causing host death or chronic infection. Here, we explored the cellular and molecular mechanisms that could explain why CD8^+^ T cell-mediated immunity during infection with the human protozoan parasite *Trypanosoma cruzi* is not optimal. For that purpose, we compared the CD8^+^ T-cell mediated immune responses in mice infected with *T. cruzi* or vaccinated with a recombinant adenovirus expressing an immunodominant parasite antigen. Several functional and phenotypic characteristics of specific CD8^+^ T cells overlapped. Among few exceptions was an accelerated expansion of the immune response in adenoviral vaccinated mice when compared to infected ones. Also, there was an upregulated expression of the apoptotic-signaling receptor CD95 on the surface of specific T cells from infected mice, which was not observed in the case of adenoviral-vaccinated mice. Most importantly, adenoviral vaccine provided at the time of infection significantly reduced the upregulation of CD95 expression and the proapoptotic phenotype of pathogen-specific CD8^+^ cells expanded during infection. In parallel, infected adenovirus-vaccinated mice had a stronger CD8 T-cell mediated immune response and survived an otherwise lethal infection. We concluded that a suboptimal CD8^+^ T-cell response is associated with an upregulation of CD95 expression and a proapoptotic phenotype. Both can be blocked by adenoviral vaccination.

## Author Summary

Killer lymphocytes are important mediators of the immunological resistance against infections caused by virus, bacteria and parasites. In some circumstances, however, these lymphocytes are unable to properly eliminate the microorganisms which survive, causing death or establishing chronic infections. The purpose of our study was to understand why these killer cells do not succeed during infection with a human protozoan parasite. For that purpose, we compared the immune responses in animals infected or vaccinated. Many characteristics of these killer cells were similar. Among few exceptions was an accelerated immune response in vaccinated animals when compared to infected ones. Also, we observed on the surface of the killer lymphocytes from infected, but not from vaccinated animals, an increased expression of a protein involved in signaling cell death. Most importantly, vaccine significantly reduced the higher expression of this cell-death receptor. In parallel, these animals had a stronger immune response and cured infection. We concluded that a deficient killer cell response observed during infection was associated with an upregulation of this cell-death receptor and it was changed by vaccination.

## Introduction

The digenetic intracellular protozoan parasite *Trypanosoma cruzi* (*T. cruzi*) is the etiologic agent of Chagas' disease, an acute and chronic illness affecting millions of individual in the Americas [1]. After contact with *T. cruzi*, humans and mice develop MHC class Ia-restricted CD8^+^ T cells specific for immunodominant parasite epitopes [2]–[7]. In highly susceptible hosts, such as A/Sn mice, the immune response is unable to control acute-phase pathology and prevent death. In most hosts, however, these specific CD8^+^ T cells are critical for survival following infection, even when limited numbers of parasites initiate infection [2], [8]. Despite the CD8^+^ T cell-mediated immune response, *T. cruzi* usually survives and establishes a life-long chronic infection. Parasite persistence is an important element of chronic-phase pathologies that occur many years or even decades after initial infection [9]–[15]. Therefore, in any circumstance, the CD8^+^ T cell-mediated immune response completely eliminate the parasite. The reason for this ineffective or suboptimal immune response is not fully understood. Several potentially significant mechanisms have been described for parasite evasion of the immune response and may account for short- and long-term parasite survival [16].

In contrast to the observations made during experimental mouse infection, immunization with adenoviral vaccines expressing a parasite immunodominant antigen can elicit a strong CD8^+^ T cell-mediated immunity against acute and late pathologies after an infectious challenge with *T. cruzi* [17]–[21]. Although it is well established that these specific CD8^+^ T cells induced by vaccination eliminate parasites, it is not clear which properties of these T cells are responsible for sustaining highly effective immunity against *T. cruzi*-induced pathology. It is paradoxical that specific CD8^+^ T cells elicited by infection or vaccination are cytotoxic *in vivo* and secrete IFN-γ, which are mechanisms that mediate protective immunity against *T. cruzi* [5], [17].

To understand this problem, we hypothesized that CD8^+^ T cells induced during infection had different functional or phenotypic properties from specific T cells generated by adenoviral vaccines. Accordingly, our approach consisted of a detailed comparison of the kinetics, function, and phenotype of the CD8^+^ T cells induced by either experimental infection or immunization with recombinant adenoviral vaccine expressing the immunodominant antigen, amastigote surface protein 2, of *T. cruzi* (AdASP-2). Essentially, our results showed that parasite infection and AdASP-2 vaccination elicited effector CD8^+^ T cells that mostly overlapped in phenotype and function. An important and recurrent exception was the overexpression of the apoptotic receptor CD95 on the surface of the specific CD8^+^ T cells observed during *T. cruzi* infection. In contrast, AdASP-2 vaccine induced T cells that failed to upregulate CD95 expression. Moreover, immunization with the AdASP-2 vaccine performed simultaneously with the infectious challenge pre-programs specific CD8^+^ T cells to mediate protective immunity. In adenoviral-vaccinated animals, protective CD8^+^ T cells that expand after an infectious challenge had reduced CD95 expression and a reduced proapoptotic phenotype. Our observation indicates that CD95 expression by suboptimal CD8^+^ T cells during infection may be one of the key factors leading to an early increase in apoptosis and a reduced immune response. Furthermore, adenoviral vaccines may pre-program pathogen-specific T cells by reducing the levels of CD95 expression and apoptosis and, therefore, promote T cell accumulation and efficacy.

## Results

### Protective CD8^+^ T cells are elicited by a single dose of AdASP-2 vaccine provided shortly before or at the same time as an infectious challenge

**Figure 1:**
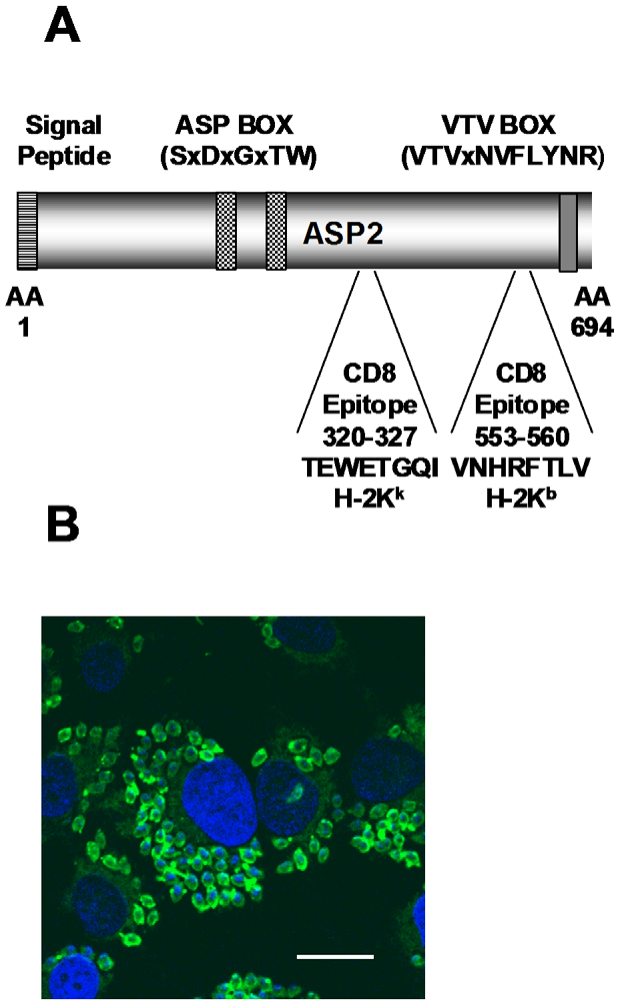
Schematic representation of the ASP-2 antigen and its expression in intra-cellular amastigotes of *T. cruzi*. **A**) The protein is a prototypical member of the *trans*-sialidase family of *T. cruzi* surface antigens containing a putative signal peptide at the N-terminal region, 2 ASP-box sequences, and a VTV-box at the C-terminal domain. The protein is attached to the membrane through a glycophosphatidylinositol anchor. **B**) ASP-2 expression in intra-cellular amastigotes was determined by immunofluorescence with the specific MAb K22. HeLa cells were infected for 48 h with trypomastigotes of the Y strain. After fixation, indirect immunofluorescence and DAPI staining were performed and imaged under fluorescence microscopy. Bar, 14 µM. Photomicrography kindly provided by Dr. Clara Claser (Singapore Immunology Network -SIgN, Singapore).

Recently, the AdASP-2 vaccine was described [17]–[21]. This vaccine expresses the immunodominant antigen ASP-2, a member of the *trans*-sialidase superfamily of surface proteins of *T. cruzi*. This antigen is abundantly expressed by intracellular amastigotes and contains 2 immunodominant epitopes (one for H-2K^k^ [TEWETGQI] and one for H-2K^b^ [VNHRFTLV]) recognized by protective CD8^+^ T cells during mouse infection with the Y strain of *T. cruzi*. (Figure 1A and B, [ref. 3], [5]).

**Figure 2:**
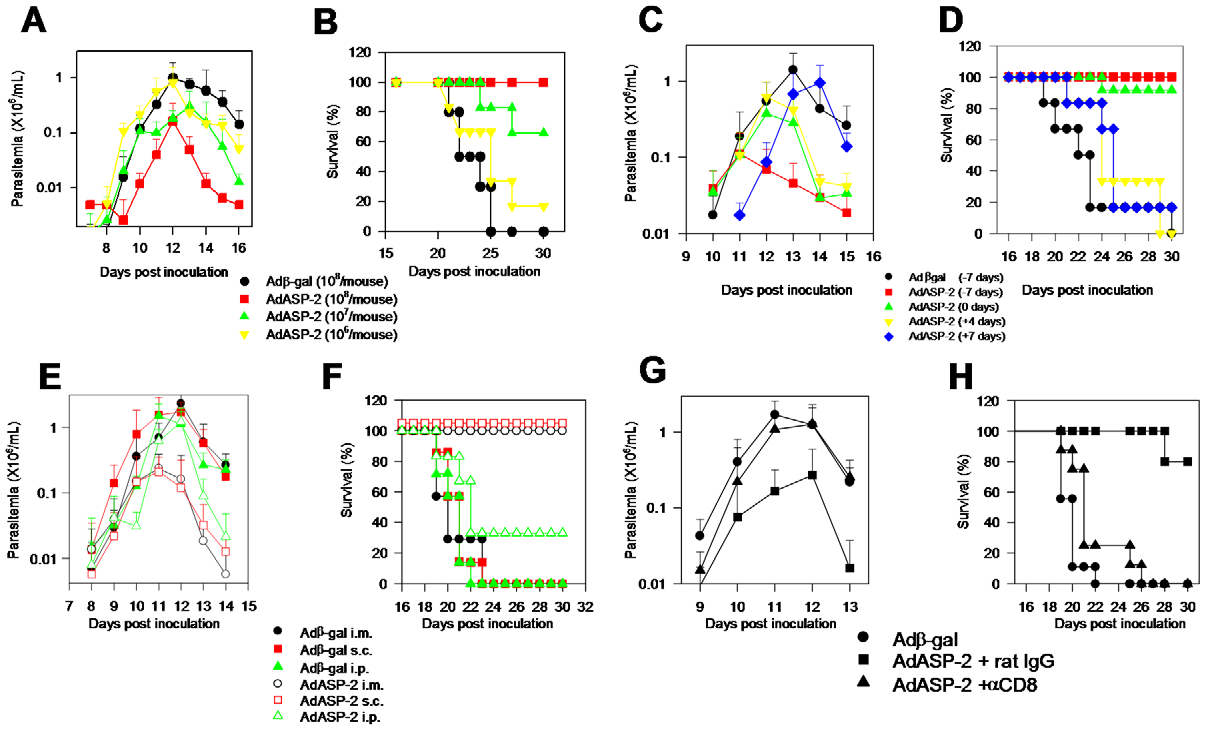
Parasitemia and mortality in A/Sn mice immunized with AdASP-2 vaccine and challenged with trypomastigotes. **A**) A/Sn mice were immunized i.m. with the indicated doses (pfu) of AdASP-2 vaccine or control Adβ-gal. Seven days later, mice were challenged s.c. with 150 bloodstream trypomastigotes of the Y strain of *T. cruzi*. Parasitemia was followed daily from days 9 to 13 after challenge. The results represent the mean ± SD values for 6 mice. At the peak parasitemia (day 11), values of mice immunized with each different dose were compared by one-way ANOVA and Tukey HSD tests and the results were as follows: **i)** Groups of mice that received doses of 10^8^ or 10^7^ pfu of AdASP-2 vaccine had values lower than the group of mice injected with Adβ-gal (*P*<0.01). **B**) Kaplan–Meier curves for survival of the different groups were compared using the logrank test. The results of the comparisons were as follows: **i)** Groups of mice injected with 10^8^ or 10^7^ pfu of AdASP-2 vaccine survived longer than the group injected with Adβ-gal (*P*<0.01). **C**) A/Sn mice were immunized i.m. on the indicated days before or after infection with AdASP-2 vaccine or control Adβ-gal (2×10^8^ pfu/mouse). All mice were challenged s.c. as described above. Although the day of the peak parasitemia was different for each group, we used the maximal values for statistical comparison. The results of the comparisons were as follows: **i)** Mice vaccinated on day −7 (red) with AdASP-2 vaccine had values lower than those of any other group. **ii)** Mice vaccinated on days 0 (green) or +4 (yellow) with AdASP-2 vaccine had values lower than the group of mice injected with Adβ-gal (black) or AdASP-2 on day +7 (blue, *P*<0.01). **D**) Kaplan–Meier curves for survival of the different groups were compared and the results were as follows: **i)** Groups of mice vaccinated with AdASP-2 vaccine on days −7 (red) or 0 (green) survived longer than the groups of mice injected with Adβ-gal (black) or AdASP-2 vaccine on days +4 (yellow) or +7 (blue) (*P*<0.01 in all cases). **E**) A/Sn mice were immunized with AdASP-2 vaccine or Adβ-gal (2×10^8^ pfu/mouse) by the indicated routes (i.m., s.c., or i.p.). All mice were challenged s.c. on the same day as the immunization, as described above. The parasitemia data for each mouse group are represented in terms of mean ± SD (n = 6). Although the day of the peak parasitemia was different for each group, we used the maximal values for statistical comparison. The results of the comparisons were that mice vaccinated i.m. or s.c. with the AdASP-2 vaccine had values lower than those of any other groups (*P*<0.01). **F**) Kaplan–Meier curves for survival of the different groups were compared and the results were as follows: **i)** Mice vaccinated i.m. or s.c. with the AdASP-2 vaccine survived longer than any other groups (*P*<0.01). **ii)** Mice vaccinated i.p. with AdASP-2 vaccine survived longer than mice that received Adβ-gal (*P*<0.05). **G**) A/Sn mice were immunized i.m. with AdASP-2 vaccine or control Adβ-gal (2×10^8^ pfu/mouse). Seven days later, mice were challenged s.c. as described above. Before and after challenge, mice were treated as described in the Methods section with rat IgG (control) or anti-CD8 MAb. The parasitemia for each mouse group is represented as mean ± SD (n = 6). Although the day of the peak parasitemia was different for each group, we used the maximal values for statistical comparison. AdASP-2-immunized mice treated with rat IgG had a significantly lower parasitemia (*P*<0.01) when compared to those of non-immune animals or AdASP-2-vaccinated mice treated with anti-CD8 MAb. **H**) Kaplan–Meier curves for survival of the different groups were compared and the results showed that AdASP-2-immunized mice treated with rat IgG survived significantly longer (*P*<0.01) than non-immune animals or AdASP-2-immunized mice treated with anti-CD8 MAb. The results described above are representative of 2 or more independent experiments.

To establish a simple method to study the induction of protective CD8^+^ T cells against *T. cruzi* infection, we immunized susceptible A/Sn mice with a single dose of AdASP-2 vaccine. As shown in Figure 2A, i.m. immunization with increasing doses of AdASP-2 (ranging from 10^6^ to 10^8^ pfu) 7 days prior to infection led to a dose-dependent reduction in peak parasitemia. Vaccination also significantly retarded or reduced mouse mortality (Figure 2B). As a control, mice injected with the higher dose of adenovirus vector expressing the β-galactosidase protein (Adβ-gal) developed higher parasitemia (Figure 2A) and died sooner than the other mouse groups following experimental challenge (Figure 2B).

From the experiment described above, we considered a single dose of ≥10^8^ pfu sufficient to protect against a challenge with *T. cruzi*. The timing of administration of the adenoviral vaccine was determined by immunizations performed from 7 days before up to 7 days after infection. As shown in Figure 2C and 2D, even when administered on the same day as the infectious challenge, immunization with AdASP-2 vaccine elicited significant protective immunity, impairing the development of parasitemia and improving mouse survival. In contrast, administration of AdASP-2 vaccine after the challenge had limited or no impact on infection.

To determine whether the route of immunization affected protective immunity, we injected AdASP-2 vaccine on the same day as infection by the i.m., s.c., or i.p routes. While mice immunized i.m. or s.c. controlled parasitemia and survived infection, animals injected by the i.p. route showed limited control of parasite development and mouse survival (Figure 2E and 2F). Finally, to evaluate the role of CD8^+^ T cells for protection, AdASP-2-immunized mice were treated with MAb to CD8 prior to infection. The depletion of CD8^+^ T cells led to a complete reversal of immunity as evaluated by parasitemia (Figure 2G) and mouse survival (Figure 2H). We concluded that a single dose of 2×10^8^ pfu of AdASP-2 vaccine administered 7 days prior to or even on the same day as the infectious challenge can be used as a simple model to elicit protective CD8^+^ T cells against an otherwise lethal infection with *T. cruzi*.

To determine whether AdASP-2 vaccination cured *T. cruzi* infection, we collected 0.5 mL of the blood 24 months after challenge and transferred i.p. to naïve A/Sn mice. Four of the 15 mice (26.66%) developed patent parasitemia and died. In contrast, 100% of control mice injected with 150 parasites developed patent parasitemia and died (data not shown). Based on that, we considered that AdASP-2 vaccination in fact cured *T. cruzi* infection in 73.33% of the vaccinated animals. The absence of infection can not be attributed to passively transferred immunity because injection of 0.5 mL of blood of vaccinated mice admixed with 100 viable bloodstream trypomastigotes also does not confer protection to naïve A/Sn (data not shown).

### Higher numbers of multifunctional specific CD8^+^ T lymphocytes are present as a result of the adenovirus vaccination

The fact that AdASP-2 vaccine administered simultaneously with the infectious challenge could provide a significant degree of protective immunity raised the possibility that vaccine- or *T. cruzi*-induced CD8^+^ T cells had different traits. To understand the cellular basis for the biological properties of the CD8^+^ T cells elicited by infection or vaccination, we compared certain functional and phenotypic profiles.

**Figure 3:**
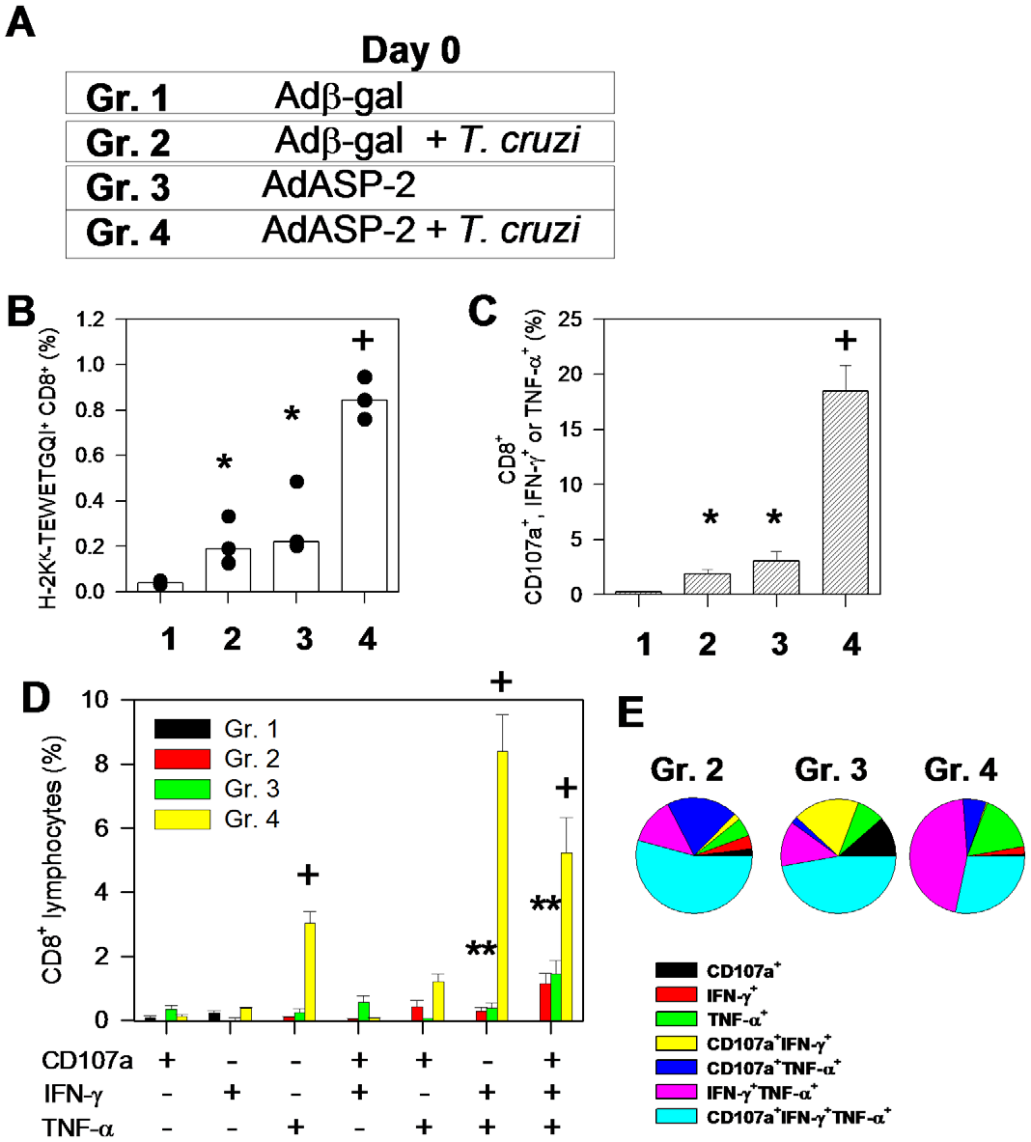
Specific CD8^+^ T cell-mediated immune responses of infected, immunized or infected AdASP-2 immunized A/Sn mice. **A**) A/Sn mice were immunized i.m. with Adβ-gal or AdASP-2 vaccine (2×10^8^ pfu/mouse). On the same day, half of the mice were challenged s.c. with trypomastigotes of the Y strain of *T. cruzi* (150 bloodstream parasites/mouse). **B**) Nineteen days after the immunization/challenge, we estimated the frequency of splenic H2K^k^-TEWETGQI^+^ CD8^+^ cells. The results are presented in terms of each mouse (dots) and medians (bars). **C**) Splenic cells were cultured in the presence of anti-CD107a and anti-CD28, with or without the peptide TEWETGQI. After 12 h, cells were stained for CD8, IFN-γ, and TNF-α. Frequencies were initially estimated for any CD8^+^ that expressed surface CD107a, IFN-γ or TNF-α after stimulation *in vitro* with peptide TEWETGQI. D) Subsequently, we estimated the subpopulations of CD8^+^ cells expressing each individual molecule or combination (surface CD107a, IFN-γ or TNF-α). The results are presented as the mean ± SD frequencies of CD8^+^ cells for 4 mice. The asterisks and crosses denote significantly higher numbers of peptide-specific cells than in the group of mice immunized with Adβ-gal or all other groups, respectively (*P*<0.05). **E**) Pie charts show the fraction of peptide-specific cells expressing the indicated molecules. The results are expressed as the mean values for 4 mice per group. The results are representative of 2 independent experiments.

Groups of A/Sn mice were immunized according to the protocol described in Figure 3A. In these experiments, vaccination and challenge were performed simultaneously to avoid any obvious boosting effect that might interfere with interpretation of the results. Our analysis of the immune response was done on day 19 post infection just prior to the Adβ-gal-immunized control mice started to die. We observed consistently similar frequencies of peptide-specific cells in mice that were infected with *T. cruzi* or vaccinated with AdASP2 (Figure 3B). Most important was the fact that significantly more peptide-specific cells were detected in infected AdASP-2- vaccinated mice. The frequency of specific CD8^+^ T cells, as determined by staining with the multimer H2K^k^-TEWETGQI, was approximately 4 times higher in infected AdASP-2-immunized mice than in mice that had only been immunized or infected (Figure 3B).

To compare important functional aspects of antigen-specific CD8^+^ T cells, we performed staining of CD8^+^ T cells following *in vitro* peptide stimulation for surface mobilization of CD107a [22], a marker for T-cell degranulation, and intracellular effector cytokines (IFN-γ and TNF-α, intra-cellular cytokine staining-ICS). Estimates of frequencies of splenic functional peptide-specific CD8^+^ cells that mobilize CD107a to their surfaces, express IFN-γ or TNF-α were 6- to 9-fold higher in infected AdASP-2-immunized mice than in the spleen of mice that had been only immunized or infected (Figure 3C). Spleen cells stimulated with the peptide TEWETGQI revealed a large fraction of multifunctional CD8^+^ cells in both, infected or immunized mice (Gr.2 and Gr. 3, Figure 3D and 3E). The analysis of the frequency of CD8^+^ T cells expressing different combinations of the effector molecules in mice infected with *T. cruzi* or immunized with AdASP-2 vaccine revealed that multifunctional triple-positive cells was the largest population in both cases, accounting for 57.36% or 47.25 % of the peptide-specific cells in infected or immunized mice, respectively. Among the double-positive cells, we found 14.15 % or 12.90 % of IFN-γ^+^/TNF-α^+^ cells in infected or vaccinated mice, respectively. The difference in terms of frequency was the higher proportion of CD107a^+^/TNF-α^+^ or CD107a^+^/IFN-γ^+^ double-positive CD8^+^ T cells in splenic cells from infected mice or immunized mice, respectively. We considered that the quality of the specific CD8^+^ T cells elicited by *T. cruzi* infection or AdASP-2 immunization generally overlapped.

The pattern of effector molecules expressed by specific CD8^+^ cells in infected AdASP-2-immunized mice retained a high frequency of triple-positive (28.28 %) and double-positive IFN-γ^+^/TNF-α^+^ cells (47.25 %) The difference we observed in this case was significantly higher frequencies of single-positive TNF-α^+^ cells in splenic cells of infected AdASP-2-immunized mice when compared to those of vaccinated or infected mice (Figure 3D and 3E).

To confirm our analyses of the immune response, we used also the C57BL/6 mouse model for experiments. Infected C57BL/6 mice do not succumb to acute infection and display a strong CD8^+^ T-cell immune response to the immunodominant epitope VNHRFTLV [2]–[4], [17], [19], [20]. After s.c. infection with 10^4^ parasites of *T. cruzi*, peak parasitemia occurred on day 8 or 9 post infection (Figure S1 A). We then followed the kinetics of the CD8 epitope-specific cells by 2 distinct assays, i.e., *in vivo* cytotoxicity and *ex vivo* surface mobilization of CD107a and ICS for IFN-γ and TNF-α, after s.c. parasite infection or i.m. immunization with AdASP-2 vaccine. We observed a more rapid *in vivo* cytotoxic response in mice immunized with the AdASP-2 vaccine when compared to the response of the infected ones (day 8, Figure S1 B). Nevertheless, by day 12 both groups had reached maximal levels. In infected mice, high levels of *in vivo* cytotoxicity were still detected by day 30. This high *in vivo* cytotoxicity is maintained for a long period of >90 days due to unknown reasons (3). Likewise, the number of CD8^+^ splenocytes that mobilize CD107a to their surfaces and express IFN-γ and/or TNF-α also appeared faster in mice immunized with the AdASP-2 vaccine than in infected mice (day 7, Figure S1 C). However, the immune response in vaccinated mice declined significantly at day 28 when compared to infected mice.

Splenic cells were collected from animals that had been infected or immunized and re-estimulated *in vitro* with the petptide VNHRFTLV. At day 7, multi-functional double positive CD107a^+^/IFN-γ^+^ and triple-positive peptide-specific CD8^+^ cells accounted for the largest populations of peptide-specific cells from immunized mice (Figure S1 D). At day 15, the multifunctional triple-positive cells accounted for the largest population in infected or AdASP-2 immunized mice (Figure S1 E). Among the double-positive cells, we found higher frequencies of IFN-γ^+^/TNF-α or CD107a^+^/IFN-γ^+^ cells in infected or vaccinated mice, respectively. A difference in terms of frequency was also observed by the higher proportion of CD107a single-positive CD8^+^ T cells in splenic cells from immunized mice (Figure S1 E).

At day 28, the frequencies of the distinct sub-populations changed little in infected mice compared to day 15. In the case of AdASP-2 immunized mice CD107a^+^/IFN-γ^+^ cells accounted for the largest population with the decline of multifunctional triple-positive cells (Figure S1 F). An increase was also seen by the higher proportion of CD107a single-positive CD8^+^ T cells in splenic cells from immunized mice.

In addition, we compared the frequencies of specific T cells in groups of mice that have been immunized and challenged according to the protocol described in Figure S2 A. On day 21, there was an increase in the frequency of peptide-specific CD8^+^ T cells from infected AdASP-2-immunized mice (Gr.4) according to the expression of effector molecules (Figure S2 B). Despite these numerical differences, the composition of the distinct T-cell subpopulation expressing one or more of these molecules was not dramatically different between infected (Gr. 2) and infected AdASP-2-immunized mice (Gr. 4, Figure S2 C and S2D). The multifunctional T cells that expressed 2 or more effector molecules accounted for more than 66% of the cells. However, differences between Gr. 2 and Gr. 4 can be seen when single-positive T cells are compared (Figure S2 C and S2 D). As we observed earlier in Figure S1 F, there was a tendency for the accumulation of more differentiated T cells (triple-positive) in infected mice (Gr. 2). Further, the distribution was more heterogeneous in the cells from AdASP-2-vaccinated mice (Gr. 3 and Gr. 4) than in the cells from infected mice (Gr. 2).

Together with the results obtained from A/Sn mice, we considered that the size and quality of the specific CD8^+^ T cells elicited by *T. cruzi* infection and AdASP-2 immunization were not dramatically different. The magnitude was similar and in terms of effector molecules, they generally overlapped at the peak of the immune response. Noteworthy were the observations that in AdASP-2 immunized mice the immune response was faster and in infected AdASP-2-immunized mice there was a significant increase in the frequency of specific CD8^+^ T cells.

A relevant question raised from these experiments was the possibility that the increase in the frequency of specific CD8^+^ T cells was due to a bystander activation caused by the AdASP-2 vaccine. To evaluate this possibility, we determined whether the frequency of CD8^+^ T cells specific for a sub-dominant epitope of *T. cruzi* was also increased in infected AdASP-2-immunized mice. For that purpose, we estimated the immune response of H-2K^b^-restricted CD8^+^ cells specific for the epitope TsKb-20. We found that AdASP-2 immunization prior to infection (Gr. 4) generated a significant increase in the frequencies of VNHRFTLV specific CD8^+^ cells as estimated by the multimer staining or by expression of IFN-γ and/or TNF-α (Figure S3 B and C, respectively). In contrast, AdASP-2 immunization prior to infection did not cause an increase in the frequency of TsKb-20 specific CD8^+^ cells when compared to mice that had been immunized with control Adβ-gal and infected with *T. cruzi* (Gr.2, Figure S3 C). In fact, the frequencies of TsKb-20 specific CD8^+^ cells were lower. This observation provided a strong argument against any bystander effect of the AdASP-2 immunization.

### Prevention of up-regulation of CD95 expression on specific CD8^+^ T cells from mice primed with the adenovirus based vaccine and simultaneously challenged with blood stream trypomastigotes

**Figure 4:**
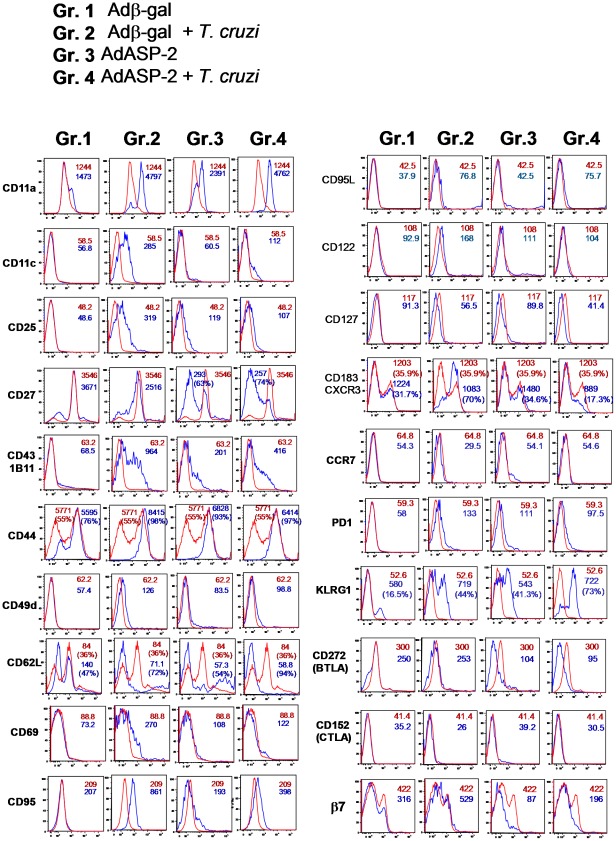
Phenotypic characterization of specific CD8^+^ T cells of infected and/or AdASP-2 immunized A/Sn mice. A/Sn mice were infected or immunized as described in the legend of Figure 3A. Splenic cells were collected nineteen days after infection/immunization. The histograms show FACS analysis on CD8^+^ cells (Gr. 1) and H-2K^k^-TEWETGQI^+^ CD8^+^ cells (Gr. 2, 3, and 4) and the indicated marker (blue). Control cells were from naive mice (red lines). Results of CD44, KLRG1 and CD183 staining are presented as MFI and frequencies of the CD44^High^, KLRG1^High^ or CD183^High^ cells, respectively. On the other hand, results of CD27 and CD62L staining are presented as MFI and frequencies of the CD27^Low^ or CD62L^Low^ cells. Representative analyses are shown from pools of cells from 3 mice. Stainings were performed 2 or more times with identical results.

Based on the fact that the magnitude and quality of the immune response was similar in infected or immunized mice, we pursued a further characterization of the phenotype of these specific cells that could account for a higher frequency of specific CD8^+^ T cells in infected AdASP-2-immunized mice. Splenic cells were stained for CD8, the H2K^k^-TEWETGQI multimer, and various T-cell surface markers. Cells were collected from animals infected, immunized, or infected-AdASP-2 immunized as described in Figure 3A. We found a similar pattern of expression for many molecules analyzed when we compared infected (Gr. 2) or AdASP-2-immunized (Gr. 3) mice. Specific CD8^+^ T cells upregulated the expression of CD11a, CD44, PD-1 and KLRG-1. On the other hand, they downregulated the expression of CD62L and CD127. Several molecules, such as CD95L, CCR7, and CTLA-4, had unchanged expression patterns when compared to those of naive CD8^+^ T cells (Figure 4).

Some molecules were expressed differently by antigen-specific CD8^+^ T cells from infected or immunized mice. CD11c, CD25, CD43 (1B11), CD69 and CD122 were expressed only on a subset of specific CD8^+^ T cells of infected mice but not on CD8^+^ T cells from immunized mice. CD27, BTLA and β7 expression were down regulated only on a subset of specific CD8^+^ T cells of immunized mice but not on CD8^+^ T cells from infected mice. To our surprise, we consistently found that the apoptotic receptor CD95 (Fas) was upregulated only on the surface of specific CD8^+^ T cells from infected but not immunized mice.

When we analyzed the phenotype of the specific CD8^+^ T cells of infected AdASP-2-immunized mice (Gr. 4), we found that they matched the ones observed on the specific CD8^+^ T cells from AdASP-2 immunized mice (Gr. 3). This observation suggests that vaccination has a dramatic influence on the phenotype of the cells expanded after the infectious challenge.

To confirm these results, we analyzed the phenotype of splenic CD8^+^ cells from immunized or infected C57BL/6 mice. Splenic cells were collected from animals that had been infected or immunized 28 or 14 days earlier, respectively, and stained for CD8, the H2K^b^-VNHRFTLV multimer and various T-cell surface markers. These days were selected because they represented the peak of their immune response (Figure S1). Overall, we found a very similar pattern of expression for most molecules analyzed. Specific CD8^+^ T cells upregulated the expression of CD11a, CD43, CD44, CD49d, CD95L, and KLRG-1. On the other hand, they downregulated the expression of CD27, CD62L, CD127, and BTLA. Several molecules, such as CD25, CD69, CCR7, and CTLA-4, had unchanged expression patterns when compared to those of naive CD8^+^ T cells (Figure S4 A).

Only a few molecules were expressed differently by antigen-specific CD8^+^ T cells from infected and immunized mice. CD11c was expressed only on a subset of specific CD8^+^ T cells of infected mice but in all CD8^+^ T cells from immunized mice. CD122 was expressed more strongly in specific CD8^+^ T cells from mice immunized with the AdASP-2 vaccine; nevertheless, the expression was still low. On the other hand, PD1, CD43 (1B11), and CD183 were upregulated only on the surface of specific CD8^+^ T cells from immunized but not infected mice (Figure S4 A).

Consistent with the results described in A/Sn infected mice, we found that the apoptotic receptor CD95 was upregulated only on the surface of specific CD8^+^ T cells from infected but not immunized mice. The kinetics of CD95 expression on the surface of specific CD8^+^ T cells of infected mice revealed that the maximum expression occurred at 14 to 28 days after infection. Thereafter, a significant decline was observed. In contrast, the expression of CD95 on specific CD8^+^ T cells from AdASP-2-immunized mice did not change during the evaluation period in comparison to that of naive CD8^+^ T cells (Figure S4 B).

MyD88 activation, IL-12 and IFN-type 1 are described as important inflammatory mediators during *T. cruzi* infection in mice [23]. Accordingly, we tested whether the upregulation of CD95 on the surface of specific CD8^+^ T cells was dependent on their expression. For that purpose, we used genetically deficient (KO) mice that failed to express MyD88, p40 (IL-12/IL-23) or IFN-1 receptor. As shown in Figure S5, specific CD8^+^ T cells from infected MyD88, IL-12/IL-23 or IFN-1 receptor KO mice upregulated CD95 on their surface at the same level as WT mice. These results suggested that upregulation of CD95 was independent of the expression of any of these molecules.

**Figure 5:**
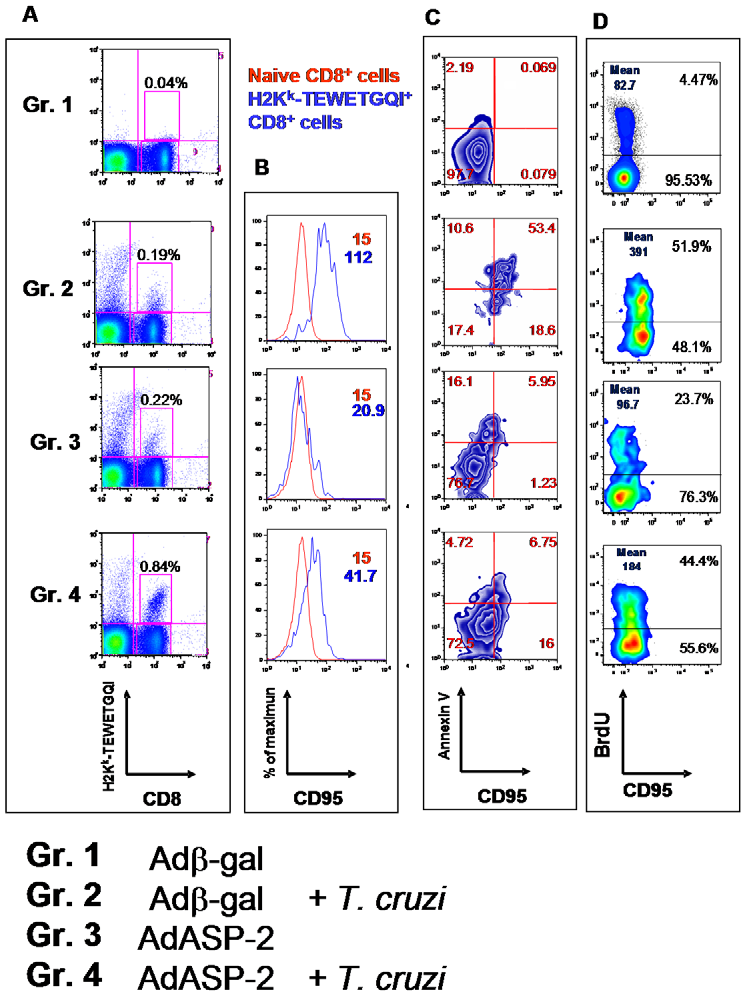
Frequency, phenotypic characterization and proliferative capacity of specific CD8^+^ T cells. The protocol of the experiment was as described in the legend of Figure 3A. **A**) Nineteen days after the immunization/challenge, we estimated the frequency of splenic H2K^k^-TEWETGQI^+^ CD8^+^ cells. FACS charts are for a representative mouse (median) from 3 mice. Numbers represent frequencies of splenic cells. **B**) Splenic cells were stained for CD8, H2K^k^-TEWETGQI and CD95 prior to analysis by FACS (blue lines). Control cells were from naive mice (red lines). **C**) The histograms show FACS analysis on CD8^+^ cells (Gr. 1) and H-2K^k^-TEWETGQI^+^ CD8^+^ cells (Gr. 2, 3, and 4) stained for CD95 and annexin V ligand. Numbers represent frequencies of CD8^+^ cells (Gr.1) or H2K^k^-TEWETGQI CD8^+^ cells (Gr. 2, 3, and 4). **D**) Mice were treated with BrdU for 4 days prior to euthanasia and splenic cells were stained for CD8, H-2K^k^-TEWETGQI, CD95 and BrdU prior to FACS analysis. The histograms show analysis on CD8^+^ cells (Gr. 1) or H2K^k^-TEWETGQI^+^ CD8^+^ cells (Gr. 2, 3, and 4) stained for CD95 and incorporated BrdU. Numbers represent frequencies of CD8^+^ cells (Gr.1) or H2K^k^-TEWETGQI CD8^+^ cells (Gr. 2, 3, and 4). Mean numbers represents MFI for CD95 staining. Representative analyses (medians) are shown for 3 mice per experiment. The experiment was performed two or more times with similar results.

The fact that specific H2K^K^-TEWETGQI^+^ CD8^+^cells expressed higher amounts of the apoptotic receptor CD95 led us to determine whether they could be indeed in a proapoptotic state. Splenic H2K^K^-TEWETGQI^+^ CD8^+^cells were analyzed in infected, vaccinated or infected AdASP-2-immunized mice 19 days after challenge/infection. We estimated their frequencies, expression of CD95 and annexin V ligand and their *in vivo* proliferative capacity. As earlier described, a significantly higher frequency of H2K^K^-TEWETGQI^+^ CD8^+^ cells was detected in the spleen of infected-AdSP-2 immunized mice (Gr. 4, Figure 5A). Also, the expression of CD95 was higher on the surface of specific CD8^+^ from infected mice (Gr. 2, Figure 5B, C and D). Most relevant was the fact that we found that a higher frequency of H2K^K^-TEWETGQI^+^ CD95^+^ CD8^+^ cells from Gr. 2 (infected) mice stained positive for annexin V (Gr. 2, Figure 5C). This frequency was significantly higher when compared to that of cells from Gr. 4 (mean values, 58.05%±6.38% and 17.29%±6.42%, n = 3, respectively; P<0.05).

To confirm that the lower frequency of specific CD8^+^ T cells was not due to a diminished proliferative capacity of cells from infected mice, we evaluated their *in vivo* proliferative capacity. We found that a similar proportion of the H2K^K^-TEWETGQI^+^ CD8^+^ cells incorporated BrdU *in vivo* in infected or infected-AdASP-2 vaccinated mice, indicating that the proliferative capacity of these cells was not significantly different (Gr. 2 and Gr. 4, Figure 5C). From these experiments, we concluded that in the highly susceptible A/Sn mice, H2K^K^-TEWETGQI^+^ CD8^+^ cells were proliferating at a similar rate in infected or infected-AdASP-2 immunized mice. Nevertheless, H2K^K^-TEWETGQI^+^ CD8^+^ cells from infected mice expressed higher levels of the apoptotic receptor CD95 and the earlier apoptotic marker ligand for annexin V.

We subsequently confirmed these observations using C57BL/6 mice. These mice were immunized and/or infected as described in Figure S2 A. As shown in Figure S6 B, although a clear pattern of upregulation of CD95 can be observed in cells from infected animals (Gr. 2), no or limited regulation were observed in cells from animals that were only immunized with AdASP-2 vaccine (Gr. 3) or from infected-AdASP-2 immunized mice (Gr. 4), respectively. Most important, we found that the frequency of H2K^b^-VNHRFTLV^+^ CD8^+^ cells from Gr. 2 (infected) mice that stained for annexin V was higher than those of the other mouse groups (Figure S6 B).

**Figure 6:**
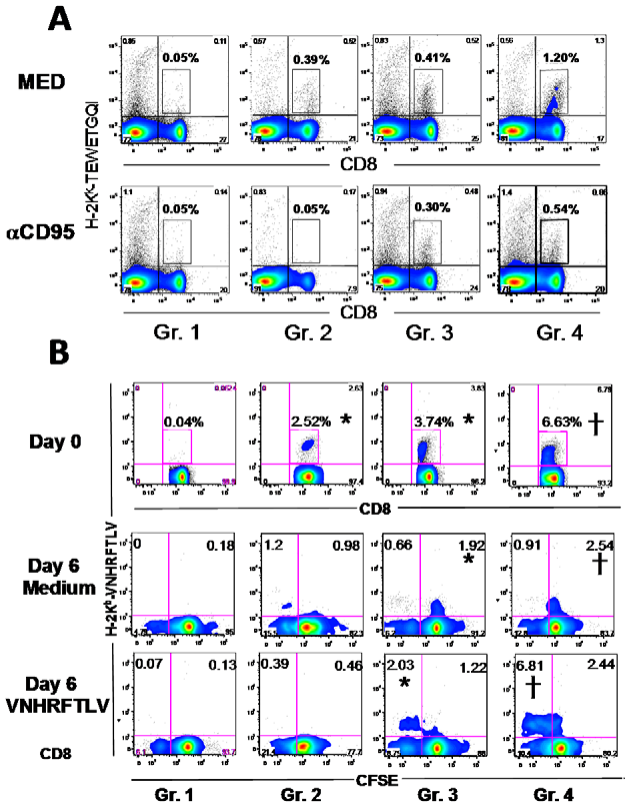
*In vitro* survival capacity of specific CD8^+^ T cells from infected and/or AdASP-2 immunized mice. **A**) A/Sn mice were immunized i.m. with Adβ-gal or AdASP-2 vaccine (2×10^8^ pfu/mouse). On the same day, half of the mice were challenged s.c. with trypomastigotes of the Y strain of *T. cruzi* (150 bloodstream parasites/mouse). Twenty days after the immunization/challenge, splenic cells were cultured in the presence of αCD95 (10 µg/mL) or Medium. After 24 h, we estimated the frequencies of splenic H2K^k^-TEWETGQI^+^ CD8^+^ cells. Results are from pools of cells from four mice. The experiment was performed twice with similar results. Mice were immunized i.m. with 2×10^8^ pfu of the recombinant adenovirus and challenged s.c. with 10^4^ trypomastigotes of *T. cruzi* as described in the Figure S2 A. Twenty one days after adenoviral immunization, splenic cells were immediately stained with anti-CD8 and H2K^b^-VNHRFTLV prior to FACS analysis (Day 0). Alternatively, they were labeled with CFSE and cultured in the absence or presence of 10 µM VNHRFTLV (Day 6). After that period, the cells were stained with anti-CD8 and H2K^b^-VNHRFTLV prior to FACS analyses. Results are from pools of cells from three mice. These mice were also analyzed individually and the results were identical. The numbers represent the frequency of CD8^+^ cells in each quadrant. The asterisks and crosses denote significantly higher numbers of peptide-specific cells than in the group of mice immunized with Adβ-gal or all other groups, respectively (*P*<0.05).

The observations that the specific CD8^+^ T cells showed a higher CD95 expression and a pro-apoptotic phenotype *in vivo* strongly suggest that these cells have a lower survival rate. Initially, we tested their behavior upon CD95 engagement. Upon *in vitro* exposure to αCD95, specific-CD8^+^ cells from infected mice (Gr. 2) survived poorly. We estimated that 88.2% of them died (Figure 6A). In contrast, under the same conditions, 73% of the cells from AdASP-2-immunized mice (Gr. 3) survived. In the case of AdASP-2-immunized and infected mice (Gr. 4), we observed that a fraction of the cells (45%) survived the exposure to αCD95. However, considering the high initial frequency of these cells, the total of 0.54% of total splenic cells was still higher than the other groups.

To test the *in vitro* proliferation capacity, we stimulated splenic cells collected from infected C57BL/6 mice with the cognate peptide VNHRFTLV. We observed that after 6 days in culture, specific CD8^+^ cells from infected mice (Gr. 2) were no longer detected (Figure 6B). In contrast, specific CD8^+^ cells from AdASP-2-immunized mice, infected (Gr. 4) or not (Gr. 3) with *T. cruzi*, were still alive. In the presence of the peptide, they also proliferated. Based on that, we conclude that, *in vitro*, the ability to survive to CD95 engagement or to survive for long periods of time even in the presence of the antigen, was strikingly different between specific CD8^+^ T cells from *T. cruzi* infected or AdASP-2 immunized mice. The possible reason why we found proliferating cells *in vivo*, but not *in vitro*, is that *in vivo*, as the cells die, they were being replenished by new ones which proliferate for few cycles. *In vitro*, as they die, they disappear.

Because experimental *T. cruzi* infection can vary widely according to the combination of mouse and parasite strain used, we attempted to reproduce some of our results using a different experimental model. For that purpose, we immunized BALB/c mice with an adenoviral vaccine expressing the *trans*-sialidase antigen of *T. cruzi* (AdTS), according to the protocol described in Figure S7 A. AdTS contains the immunodominant H-2K^d^-restricted epitope, IYNVGQVSI [2], [3], [5], [7]. Immunization with AdTS vaccine significantly reduced the parasitemia caused by parasites of the Brazil strain (Figure S7 B). On day 28 post infection, we again observed a higher frequency of specific cells in mice simultaneously immunized with AdTS vaccine when compared to those in the other mouse groups. The frequency of specific CD8^+^ cells as determined by staining with the multimer H-2K^d^-IYNVGQVSI was 3–4 times higher in infected AdTS-immunized mice than in mice that had only been infected or immunized (Figure S7 C). Estimates of frequencies of peptide-specific CD8^+^ cells by ICS assays also detected 6.89- or 18.92-times higher CD107a- and IFN-γ- and/or TNF-α-positive cells in infected AdTS-immunized mice than in infected or immunized mice, respectively (Figure S7 D).

To extend the observations for CD95 expression by specific CD8^+^ T cells, we compared the phenotypes of H2K^d^-IYNVGQVSI^+^ CD8^+^ cells from the different mouse groups. A clear pattern of upregulated expression of CD95 was observed in specific CD8^+^ T cells from infected animals (Gr. 2, Figure S8 B). In contrast, cells collected from mice immunized with the AdTS vaccine (Gr. 3) did not modulate CD95 expression. H2K^d^- IYNVGQVSI^+^ CD8^+^ cells from mice immunized with the AdTS vaccine before challenge expressed significantly lower levels of CD95 than those of specific CD8^+^ T cells from infected animals (Gr. 2, Figure S8 B). This different pattern was not observed when we estimated the expression of KLRG1 and CD95L on the surface of these same cells (Figure S8 C and D, respectively). The difference is also observed after different days of infection (Figure S8 E). We concluded from these experiments that *T. cruzi* infection with 2 distinct strains of the parasite leads to an upregulation of CD95 expression on parasite-specific CD8^+^ T cells. On the other hand, immunization with distinct adenoviral vaccines does not modulate CD95 expression on transgene-specific CD8^+^ T cells. Finally, priming with the adenoviral vaccines prior to or simultaneously with the challenge partially prevents aberrant CD95 expression on parasite-specific CD8^+^ T cells in these different infection models.

## Discussion

In the present study, we investigated the cellular and molecular mechanisms that may account for the suboptimal immunity of specific CD8^+^ T cells generated during infection with *T. cruzi* in mice. Accordingly, our strategy was to compare the suboptimal immune response elicited by parasite infection with the response elicited by a highly effective adenoviral vaccine expressing the parasite's immunodominant epitopes. Our main hypothesis was that CD8^+^ T cells induced by *T. cruzi* infection had one or more properties not present in cells expanded by adenoviral vaccines, and this caused a suboptimal immune response.

A detailed comparison of the specific CD8^+^ T cells elicited by infection or vaccination revealed that, in general, the cells overlapped in their functional or phenotypic characteristics. Epitope-specific CD8^+^ T cells were highly cytotoxic *in vivo* and secreted IFN-γ, a cytokine that is critical for control of *T. cruzi* multiplication [3], [17]. Further, these cells mobilize CD107a to their surface after *in vitro* stimulation with the cognate peptide, indicating that a large proportion of these cells are prepared for granule exocytosis. This observation is supported by cytotoxicity experiments showing that the specific T cells are highly cytotoxic *in vivo*. Analysis of the expression of IFN-γ and TNF-α by ICS also revealed that after experimental infection or immunization with AdASP-2 vaccine, the majority of specific T cells were a population of multifunctional T cells that mobilized surface CD107a and expressed both cytokines. Other cytokines evaluated, such as IL-2 or IL-10, were not detected by ICS (data not shown, [18]).

Similarly to the functional aspects described above, the surface phenotype of the specific CD8^+^ T cells was characteristic of T_effector_ cells. The fact that effector functions of CD8^+^ T cells elicited by *T. cruzi* infection overlap with those elicited by the AdASP-2 vaccine emphasizes that these cell functions may be important for control of the pathogen multiplication and, in the case of the most mouse strains, the acute-phase pathology.

A clear difference we observed between the immune responses elicited by *T. cruzi* infection or AdASP-2 vaccine was in the kinetics of the immune response. The generation of cytotoxicity and IFN-γ-producing cells occurred faster in mice immunized with the AdASP-2 vaccine than in those infected with *T. cruzi* a fact previously described by us and others [2], [3], [4], [24]. The precise reasons for the delay in the immune response after *T. cruzi* challenge are debatable and certainly may be important for the parasite to establish a successful infection. In earlier studies, the timing for the development of the T-cell immune response was critically controlled by the parasite load [2], [4], [24]. Because parasites contain antigen and, possibly, yet to be identified adjuvant molecules, the effect of the parasite load could be associated with either one individually, or both. Padilla et al. [24] proposed that the delay was not due to the absence of parasite antigen but to the absence of sufficient parasite TLR agonists considered as *T. cruzi* adjuvant molecules. We question this interpretation because, recently, we described that the development of the CD8^+^ T cell immune response during experimental *T. cruzi* infection progressed well in genetically deficient mice that do not express TLR-2, TLR-4, TLR-9, or even the adaptor molecule MyD88 [25]. Further, even when the host immune response was accelerated by the addition of TLR agonists, no changes in the acute-phase pathology were described [24]. Therefore, other intrinsic characteristics of the CD8^+^ T cells may explain the difference in the timing of specific CD8^+^ T-cell activation.

In accord with this possibility, an important observation from our study was that epitope-specific CD8^+^ T cells expanded during infection expressed higher levels of CD95 than naive CD8^+^ cells or epitope-specific cells from mice immunized with adenoviral vaccines. This phenomenon occurred in different mouse strains (A/Sn, C57BL/6 and BALB/c) infected with 2 distinct parasite isolates (Y and Brazil). This aspect is important because in many cases there may be differences when using different parasites isolates in terms of mechanism of immunity activated during infection [26]. This aberrant pattern of upregulated expression was the only one consistently observed when compared to any other surface adhesion/homing/activation receptor that we analyzed. In addition to the specific CD8^+^ T cells expressing higher levels of CD95, CD95^hi^ cells stained positively for annexin V, a marker for apoptotic cells. Compatible with an increased apoptosis rate, we found that specific CD8^+^ T cells accumulated less *in vivo*. These smaller numbers cannot be explained by lower proliferation rates. *In vivo* incorporation of BrdU indicated similar proliferation rates of cells from infected and infected AdASP-2-vaccinated mice.

CD95 functions during the immune response are multiple and still the subject of intense studies [27]–[29]. The presence of low levels of CD95L on the surface of antigen-presenting cells (APCs) may serve as a co-stimulatory signal for naive CD95-expressing T cells [30]. On the other hand, higher levels of the ligand provide a negative signal for these same cells by blocking their activation through the CD3 complex [30], [31]. The presence of CD95L on the surface of pathogen-infected APCs has been suggested as a possible mechanism to downmodulate the immune response [31]. We could not find evidence of this theory as we could not detect CD95L on the surface of dendritic cells infected with *T. cruzi* (J. Ersching and MMR, unpublished results).

Because CD95 is an important initiator of the intrinsic pathway of apoptosis in T lymphocytes, later it cooperates with Bim in retraction of the T-cell immune response [32]–[34]. The observation that specific CD8^+^ T cells undergo modulation of CD95 expression after infection was described in specific CD8^+^ T cells following mouse infection with LCMV or in individuals infected with HIV. In the case of LCMV infection, lower levels of CD95 expression on specific CD8^+^ T cells located in the peripheral organs is associated with an increased resistance to activation-induced cell death *in vitro* [35]. In the case of human HIV infection, higher expression of CD95 leads to increased susceptibility to CD95/CD95L-mediated apoptosis and may compromise the immune response of specific CD8^+^ T cells [36].

Upregulation of CD95 expression was also described in splenic T cells during acute infection with certain *T. cruzi* strains [37]. Most relevant biologically was that in this model of infection, *in vivo* injection of antibody to CD95L, which blocks the interaction with CD95, reduced apoptosis, improved type-1 immune responses, and reduced the infection severity as estimated by parasitemia [37]. Similar treatment with anti-CD95L or anti-PD1 during infection of the highly susceptible A/Sn mice with parasites of the Y strain of *T. cruzi* did not reduce the parasitemia or the mortality, indicating that these treatments alone cannot substitute for immunization with AdASP-2 vaccine (AFA and MMR, unpublished results). The reasons for the failure observed with the anti-CD95L treatment are not clear. However the fact that CD95 is expressed in a number of different cell types which play a diverse role during the immune response may account for the difficulty to block solely the interaction of CD95 expressed on the surface of specific-CD8^+^ T cells.

The precise reason(s) why CD95 is differentially modulated after infection with *T. cruzi* or adenoviral vaccination is unknown at present. Herein, we tested whether 2 important mediators of inflammation during *T. cruzi* experimental infection (MyD88, IL-12 and IFN type I) could account for this pattern of CD95 expression on specific CD8^+^ T cells. Our results demonstrated that during infection the CD95 upregulation process was independent of the expression of each one of these mediators individually. Because these molecules are involved directly or indirectly with most cytokines that are largely produced during *T. cruzi* infection, these results may indicate that the signal for CD95 up-regulation may be given by direct interaction between APC, CD8 and CD4.

To date and to our knowledge, generally the cellular and molecular mechanisms modulating CD95 expression on lymphocytes have been poorly explored. Cytokines IL-6 and type I IFN were described as factors that either downregulate or upregulate CD95 expression, respectively [38], [39]. The environment of *T. cruzi* infection is relatively poor in IL-6 but rich in type 1 IFN [40]–[42]. It is possible that this imbalance favors the expression of CD95. On the other hand, the *in vivo* environment following immunization with human adenovirus (including recombinant type 5) is rich in type 1 IFN and IL-6. This balance may block the upregulation of CD95 [43].

A very recent observation also correlated a defective CD8^+^ T cell-mediated anti-tumor immune response with the aberrant expression of CD95 and PD1 by these cells. This pattern of CD95 expression by CD8^+^ T cells was reproduced by stimulating transgenic CD8^+^ T cells *in vitro* with immature dendritic cells pulsed with the cognate peptide [44]. Whether immature or *T. cruzi*-infected dendritic cells also have the ability to upregulate the CD95 expression of CD8^+^ T cells *in vivo* is an interesting possibility that should be evaluated. Finally, cyclon, a newly identified cytokine-inducible protein produced by T cells upon TCR activation, has been shown to exert a key role in the process of CD95 expression [45]. Whether cyclon expression following TCR activation plays a role in our system also remains to be determined.

The fact that AdASP-2 vaccine fails to upregulate CD95 and changes the program of CD8^+^ T cells expanded after an infectious challenge is unexpected but may be desirable for an efficient immune response. The lower levels of CD95 expressed would render these cells resistant to parasite-induced apoptosis. In fact, we observed that adenoviral-induced CD8^+^ T cells of multiple specificities were not affected by ongoing *T. cruzi* infection (JRV and MMR, unpublished observations). These observations disfavor the hypothesis that cytokines generated during infection can nonspecifically trigger upregulation of the CD95 molecule on activated CD8^+^ T lymphocytes.

**Figure 7:**
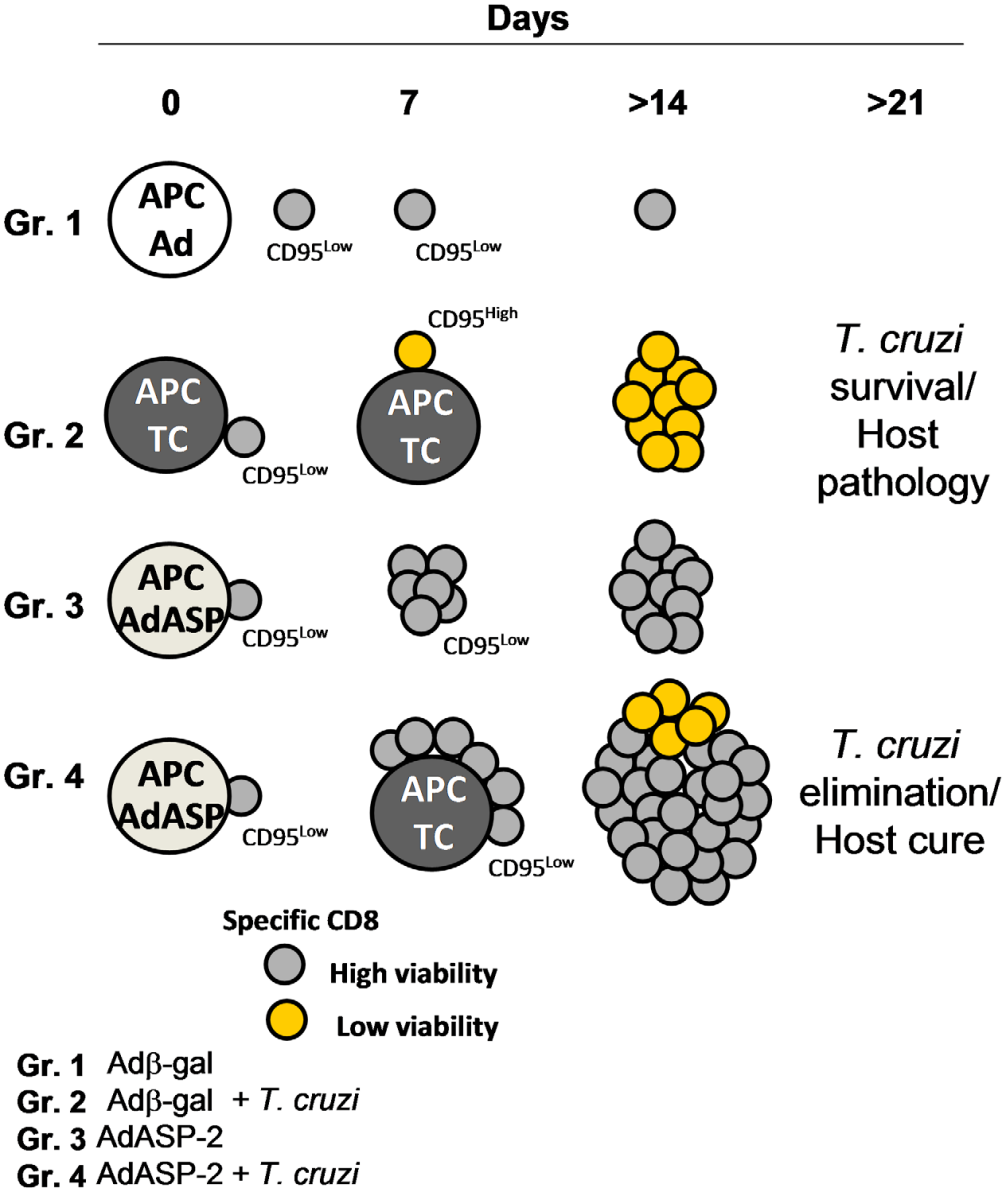
Proposed model for the differences in activation of specific CD8^+^ T cells. The initial contact of specific CD8+ T cells with APC loaded with *T. cruzi* antigen leads to a molecular program characterized by a higher expression of CD95 and a lower viability (Gr. 2). Due to the low viability of theses specific CD8^+^ T cells, optimal immune response are not generated and host pathology is developed leading to death or chronic infection. In contrast, contact of APC loaded with AdASP-2 antigens leads to a molecular program of priming and expansion of specific CD8^+^ T cells (Gr. 3). Upon a second contact with *T. cruzi*, primed CD95Low specific CD8^+^ T cells proliferate intensively and display a significantly higher viability (Gr. 4). This accelerated proliferation of highly viable CD8+ T cells lean the equilibrium towards an efficient host immune response (cure) and parasite elimination.

Based on our observations, we propose a model depicted in Figure 7. The initial contact of specific CD8^+^ T cells with APC loaded with *T. cruzi* antigen will lead to a molecular program characterized by a higher expression of CD95 and a lower viability (Gr. 2, *T. cruzi* infected mice). The low viability of specific CD8+ T cells will preclude optimal immune response. Host pathology is developed that may lead to death or chronic infection.

As oppose, initial contact with APC loaded with AdASP-2 antigens will lead to different molecular program of priming and expansion of specific CD8^+^ T cells (Gr. 3, AdASP-2 immunized mice). Upon a second contact, primed CD95^Low^ specific CD8^+^ T cells will proliferate intensively and display a significantly higher viability (Gr. 4, AdASP-2 immunized and infected mice). This accelerated proliferation coupled to a high viability would lean the equilibrium towards an efficient host immune response and parasite elimination.

The absence of an aberrant CD95 expression not only facilitates the development of a protective immune response by slowing the rate of apoptosis but it also reduces the formation of apoptotic bodies. Apoptotic bodies are described as powerful immune modulators during *T. cruzi* infection. Binding of apoptotic lymphocytes to αVβ3 expressed by macrophages leads to PGE2 and TGF-β production by macrophages, followed by the induction of ornithine decarboxylase and the synthesis of putrescine, which function as growth factors for intracellular forms of *T. cruzi* [46]–[48].

The reduction of CD95 expression on the surface of specific CD8^+^ T cells expanded following pathogen challenge may be important not only in our infection model but also in vaccination against other infectious diseases. As mentioned above, during HIV/SIV infection, CD8^+^ T cells upregulate CD95 expression [35], [49], [50]. Blocking the interaction of CD95/CD95L *in vivo* by treatment with anti-CD95L antibody preserved memory lymphocytes and cell-mediated immunity in SIV-infected primates [51]. Further, by down-modulating CD95 expression, adenoviral vaccines may reduce the formation of apoptotic bodies. As in the case of *T. cruzi* infection, these apoptotic bodies may fuel viral growth [52].

The possibility that vaccination may selectively alter specific CD8^+^ T cell phenotype and change the expression of surface receptors is only now beginning to be explored. Recently, it was described in humans that during immune responses to melanoma tumors, antigen-specific CD8^+^ T cells maintained high expression levels of the inhibitory receptor B and T lymphocyte attenuator (BTLA). Multiple vaccination doses with peptides representing CD8 epitopes in the presence, but not in the absence, of the TLR-9 agonist CpG ODN led to progressive downregulation of BTLA *in vivo*, increased resistance to BTLA-mediated inhibition, and improved T cell function [53]. This study provides important evidence that vaccination can modify antigen-specific CD8^+^ T cells, thereby qualitatively improving their function. Further, by using this same adjuvant (a TLR-9 agonist), Muraoka *et al.* [44] modified a defective CD8^+^ T cell-mediated anti-tumor immune response to a productive one by counteracting the aberrant expression of CD95 and PD1 on CD8^+^ T cells.

We consider that these results may facilitate understanding of the host–parasite relationship and present some new interpretations of the functions of T-cell vaccines. Recombinant replication-defective human and simian adenoviral vectors have proven successful against a variety of experimental infections, such as SIV, TB, malaria, toxoplasmosis, Marburg virus infection, and Ebola [54]–[69]. Nonetheless, the precise reason for their success is still unknown. By understanding the potential of these vectors to elicit an immune response and simultaneously to modulate acquired immune responses in the host, it might be possible to design simpler and more effective vaccine formulations.

## Materials and Methods

### Ethics statement

This study was carried out in strict accordance with the recommendations in the Guide for the Care and Use of Laboratory Animals of the Brazilian National Council of Animal Experimentation (http://www.cobea.org.br/). The protocol was approved by the Committee on the Ethics of Animal Experiments of the Institutional Animal Care and Use Committee at the Federal University of Sao Paulo (Id # CEP 0426/09).

### Mice and parasites

Female 5- to 8-week-old A/Sn, C57BL/6, BALB/c, p40-deficient (IL-12/IL-23 KO, [70]), MyD88- (MyD88 KO, [71]) and Interferon-I receptor (IFN-I rec KO, [72])-deficient mice were purchased from the University of São Paulo. Parasites of the Y or Brazil strain of *T. cruzi* were used in this study [2], [3]. Bloodstream trypomastigotes were obtained from mice infected 7 to 28 days earlier with parasites of the Y or Brazil strain, respectively. After estimating the parasite concentration, the blood was diluted in PBS to the desired concentration. Each mouse was inoculated with 150 trypomastigotes (A/Sn), 10^4^ trypomastigotes (C57Bl/6), or 10^3^ trypomastigotes (BALB/c) diluted in 0.2 mL PBS and administrated subcutaneously (s.c.) in the base of the tail.

### Monitoring the presence of persisting bloodstream trypomastigotes

Parasite development was monitored by counting the number of bloodstream trypomastigotes in 5 µL of fresh blood collected from the tail vein. Mouse survival was recorded daily. Twenty four months after challenge, the parasitemia was undetectable. To evaluate whether there was still a very small amount of viable circulating parasites, we collected 0.5 mL of the blood and transferred i.p. to naïve highly susceptible A/Sn mice.

### Peptide synthesis

Synthetic peptides were purchased from Genscript (Piscataway, New Jersey). Peptide purity was higher than 90%. Peptide identities were confirmed by a Q-TOF Micro equipped with an electrospray ionization source (Micromass, UK). The immunodominant epitopes of ASP-2 and TS were represented by AA VNHRFTLV and AA IYNVGQVSI, respectively. The sub-dominant epitope TsKb-20 was represented by AA ANYKFTLV (purity 99.7%).

The pentamer H2K^b^-VNHRFTLV was purchased from ProImmune Inc. (Oxford, UK). The dextramers H2K^k^-TEWETGQI and H2K^d^-IYNVGQVSI were purchased from Immudex (Copenhagen, Denmark).

### Adenoviruses used for immunization

Recombinant human type 5 replication-defective adenoviruses expressing *T. cruzi* Amastigote Surface Protein-2 (AdASP-2) or TS (AdTS) were generated, characterized, grown, and purified as previously described [17]. Mice were inoculated intramuscularly (i.m.) in each *tibialis anterior* muscle with 50 µL of viral suspension containing the indicated plaque forming units (pfu). In some experiments mice were inoculated by the s.c. route at the base of the tail. Immunological assays were performed at the indicated days after viral inoculation.

### Immunological interventions and readout assays

*In vivo* depletion of CD8^+^ T cells was performed by treating vaccinated A/Sn mice with 53.6.7 MAb. On days −6, −4, and −2 and before challenge with trypomastigotes, mice were injected i.p. with a dose of 0.5 mg of anti-CD8 or control rat IgG. Seven days after challenge, each mouse received one more dose of 0.5 mg of anti-CD8 or rat IgG. The efficacy of depletion of CD8^+^ spleen cells before challenge was more than 96% in anti-CD8-treated mice compared to that of the rat IgG-treated ones (data not shown).

For the surface mobilization (expression) of CD107a and the intracellular expression of cytokines (IFN-γ and TNF-α, ICS), splenocytes collected from A/Sn, C57BL/6 or BALB/c mice were treated with ACK buffer (NH_4_Cl, 0.15 M; KHCO_3_, 10 mM; Na_2_-EDTA 0.1 mM; pH = 7.4) for lysing the erythrocytes. Surface mobilization of CD107a and ICS were evaluated after *in vitro* culture of splenocytes in the presence or absence of the antigenic stimulus. Cells were washed 3 times in plain RPMI and re-suspended in cell culture medium consisting of RPMI 1640 medium, pH 7.4, supplemented with 10 mM Hepes, 0.2% sodium bicarbonate, 59 mg/L of penicillin, 133 mg/L of streptomycin, and 10% Hyclone fetal bovine sera (Hyclone, Logan, Utah). The viability of the cells was evaluated using 0.2% trypan blue exclusion dye to discriminate between live and dead cells. Cell concentration was adjusted to 5×10^6^ cells/mL in cell culture medium containing anti-CD28 (2 µg/mL), brefeldin A (10 µg/mL), monensin (5 µg/mL), and FITC-labeled anti-CD107a (Clone 1D4B, 2 µg/mL; BD Pharmingen). In half of the cultures, a final concentration of 10 µM of the VNHRFTLV, TEWETGQI or IYNVGQVSI peptide were added. The cells were cultivated in flat-bottom 96-well plates (Corning) in a final volume of 200 µL in duplicate, at 37°C in a humid environment containing 5% CO_2_. After 12-h incubation, cells were stained for surface markers with PerCP- and PE-labeled anti-CD8, on ice for 20 min. To detect IFN-γ and TNF-α by intracellular staining, cells were then washed twice in buffer containing PBS, 0.5% BSA, and 2 mM EDTA, fixed in 4% PBS-paraformaldehyde solution for 10 minutes, and permeabilized for 15 minutes in a PBS, 0.1% BSA, and 0.1% saponin solution. After being washed twice, cells were stained for intracellular markers using APC- and PE-labeled anti-IFN-γ (Clone XMG1.2) and PE-labeled anti-TNF-α (clone MP6-XT22), for 20 minutes on ice. Finally, cells were washed twice and fixed in 1% PBS-paraformaldehyde. At least 300,000 cells were acquired on a BD FacsCanto flow cytometer and then analyzed with FlowJo.

For the *in vivo* cytotoxicity assays, splenocytes collected from naive BALB/c or C57BL/6 mice were treated with ACK buffer (NH_4_Cl, 0.15 M; KHCO_3_, 10 mM; Na_2_-EDTA 0.1 mM; pH = 7.4) for lysing the erythrocytes. These cells were divided into 2 populations and labeled with the fluorogenic dye carboxyfluorescein diacetate succinimidyl diester (CFSE; Molecular Probes, Eugene, Oregon, USA) at a final concentration of 5 µM (CFSE_high_) or 0.5 µM (CFSE_low_). CFSE_high_ cells were coated for 40 min at 37°C with 1 µM of H-2K^b^ ASP-2 peptide VNHRFTLV. CFSE_low_ cells remained uncoated. Subsequently, CFSE_high_ cells were washed and mixed with equal numbers of CFSE_low_ cells, and 30 to 40×10^6^ total cells per mouse were injected intravenously (i.v.). Recipient animals were mice previously immunized with adenoviruses. Spleen cells of recipient mice were collected 4 h or 20 h after transfer as indicated on the legend of the figures, fixed with 1.0% paraformaldehyde and analyzed by flow cytometry by using a FacsCanto flow cytometer (BD, Mountain View, CA). The percentage of specific lysis was determined using the following formula:
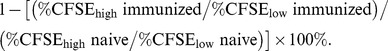



For flow cytometry analyses, we used mouse splenocytes treated with ACK buffer. Single-cell suspensions were washed in PBS, stained for 10 min at RT with biotinylated MHC I multimer H-2K^b^-VNHRFTLV, and stained for 20 min at 4°C with avidin-APC- and PerCP-labeled anti-CD8 antibodies (both from BD Pharmingen). Alternatively, splenic cells were stained for 10 min at RT with H2K^k^-TEWETGQI and H2K^d^-IYNVGQVSI dextramers (both APC-labeled) and PerCP-labeled anti-CD8 antibody. For the analyses of other cell-surface markers, single-cell suspensions from spleens of mice were stained with multimers and PerCP-labeled anti-CD8 antibody as described above. Then, FITC-labeled anti-CD11a (clone 2D7), anti-CD11c (clone HL3), anti-CD25 (clone 7D4), anti-CD31 (clone MEC13.3), anti-CD43 (clone S7), anti-CD44 (clone IM7), anti-CD49d (clone R-12), anti-CD62L (clone MEL-14), anti-CD69 (clone H1.2F3), anti-CD95 (clone Jo2), anti-CD122 (clone TM-β1), anti-CD127(clone SB/199), anti-CD95L (MFL3), anti-BTLA (8F4), anti-PD1 (J43), anti-CTLA4 (UC10-4B9), and anti-CCR-7 (4B12) (all from BD Pharmingen). Anti-CD27 (clone LG.7F9) and anti-KLRG-1 (clone MAFA) were purchased from eBioscience (San Diego, CA). Anti-CD43 (1B11) and anti-CD183 (CXCR3-173) were purchased from Biolegend (San Diego, CA). Annexin-V-PE staining was performed with a kit according to the manufacturer's instructions (BD Pharmingen). For detection of BrdU, mice were injected i.p. with 2 mg of BrdU (Sigma) for 4 days before euthanasia. The cells were treated according to the manufacturer's kit instructions and stained with anti-BrdU-APC (BD Pharmingen). At least 100,000 cells were acquired on a BD FacsCanto flow cytometer and then analyzed with FlowJo (Tree Star, Ashland, OR).

*In vitro* survival assay were performed by culturing the A/Sn splenic cells collected 20 days after inoculation of parasites or adenoviruses for 24 h in the presence or absence of anti-CD95 (clone Jo2, 10 µg/mL). Cell culture medium contained murine recombinant IL-2 (Sigma, 0.1 U/mL) and 10^6^ cells/0.2 mL. After this period, cells were stained with anti-CD8 and dexamers H-2K^k^-TEWETGQI before FACS analyses.

Alternatively, C57BL/6 splenic cells were collected from mice at the indicated days post immunization and/or challenge. These cells were labeled with 10 µM of CFSE as described above and 10^6^ cells/0.2 mL maintained for 6 days in culture medium containing or not the peptide VNHRFTLV (10 µM). After this period, cells were stained with anti-CD8 and pentarmers H-2K^b^-VNHRFTLV before FACS analyses.

### Statistical analysis

The values of parasitemia of each individual mouse were log transformed before being compared by one-way ANOVA followed by Tukey Honesty Significance Difference tests, available at the following site: http://faculty.vassar.edu/lowry/VassarStats.html. The logrank test was used to compare mouse survival rates after challenge with *T. cruzi*. The differences were considered significant when the *P* value was <0.05.

## Supporting Information

Figure S1 **Parasitemia and kinetics of specific CD8^+^ T cell-mediated immune responses during infection or vaccination.** **A**) Parasitemia of C57BL/6 mice infected s.c. with 10^4^ bloodstream trypomastigotes of *T. cruzi* **B**) Mice were infected s.c. or not with *T. cruzi* trypomastigotes as described above. In parallel, mice were immunized i.m. with AdASP-2 vaccine (2×10^8^ pfu/mouse). At the indicated days, the *in vivo* cytotoxic activity against target cells coated with peptide VNHRFTLV was determined as described in the Methods Section. The results represent the mean ± SD values for 4 mice per group. The results are representative of 3 independent experiments. **C–F**) C57BL/6 mice were infected or immunized as described above. Control mice were either naive mice or mice immunized with Adβ-gal (2×10^8^ pfu/mouse). At the indicated days after infection or immunization, these mice had their splenic cells cultured in the presence of anti-CD107a and anti-CD28, with or without the peptide VNHRFTLV. After 12 h, cells were stained for CD8, IFN-γ, and TNF-α. Frequencies were estimated for CD8^+^ cells expressing the indicated molecules after stimulation *in vitro* with peptide VNHRFTLV. The results are expressed as the mean ± SD values for 4 mice per group. The values of cultures stimulated with peptide VNHRFTLV were always subtracted from those of cultures with medium alone. Pie charts show the fraction of peptide-specific cells expressing the indicated molecules. The results are expressed as the mean values for 4 mice per group. The asterisks and crosses denote significantly higher numbers of peptide-specific cells than in the group of mice immunized with Adβ-gal or all other groups, respectively (*P*<0.05).
(PPT)

Figure S2 **Specific CD8^+^ T cell-mediated immune responses of infected, immunized or infected AdASP-2 immunized C57BL/6 mice.** **A**) C57BL/6 mice were immunized i.m. with 2×10^8^ pfu/mouse of the indicated recombinant adenovirus. Seven days later, half of the mice were challenged s.c. with 10^4^ trypomastigotes of the Y strain of *T. cruzi*. **B**) Twenty one, days after immunization with recombinant adenovirus, the splenic cells of these mice were cultured in the presence of anti-CD107a and anti-CD28, with or without the peptide VNHRFTLV. After 12 h, cells were stained with anti-CD8, anti-IFN-γ, and anti-TNF-α. The results are expressed as the total frequency of CD8^+^ cells stained for any of the indicated molecules (mean ± SD values for 4 mice per group). The values of cultures stimulated with peptide VNHRFTLV were always subtracted from those of cultures with medium alone. **C**) The same as described above except that the results are expressed as the frequencies of the indicated subpopulation of CD8^+^ cells stained for CD107a, IFN-γ, and TNF-α (mean ± SD values for 4 mice per group). **D**) Pie charts show the fraction of peptide-specific cells expressing the indicated molecules. The results are expressed as the mean values for 4 mice per group. The asterisks and crosses denote significantly higher numbers of peptide-specific cells than in the group of mice immunized with Adβ-gal or all other groups, respectively (*P*<0.05).
(PPT)

Figure S3 **Epitope-specific CD8^+^ T cell-mediated immune responses of infected and/or immunized immunized C57BL/6 mice.** **A**) C57BL/6 mice were immunized i.m. with 2×10^8^ pfu/mouse of the indicated recombinant adenovirus. Seven days later, half of the mice were challenged s.c. with 10^4^ trypomastigotes of the Y strain of *T. cruzi*. **B**) Twenty seven days after immunization with recombinant adenovirus, we estimated the frequency (%) of splenic H2K^b^-VNHRFTLV^+^ CD8^+^ cells. The results represent a pool of cells from 4 mice per group. **C**) Twenty seven days after immunization with recombinant adenovirus, the splenic cells of these mice were cultured in the presence of anti-CD28, with or without the peptide VNHRFTLV or TsKb-20. After 12 h, cells were stained with anti-CD8, anti-IFN-γ, and anti-TNF-α. The results are expressed as the total frequency (%) of CD8^+^ cells stained for any of the indicated molecules (mean ± SD values for 4 mice per group). The asterisks denote significantly higher numbers of peptide-specific cells in Gr. 4 when compared to the same sample of Gr. 2 (*P*<0.05).
(PPT)

Figure S4 **Phenotypic characterization of specific CD8^+^ T cells induced by** ***T. cruzi*** **infection or AdASP-2 immunization.** C57BL/6 mice were infected or immunized as described in the legend of Figure 2. Control mice were naive mice. **A**) Twenty-eight or 14 days after infection or immunization, respectively, these mice had their splenic cells stained with anti-CD8, H2K^b^-VNHRFTLV, and the indicated marker-specific antibody labeled prior to analysis by FACS. The histograms show the expression of the markers on H2K^b^-VNHRFTLV^+^ CD8^+^ T cells (green lines) or control naive CD8^+^ spleen cells (red lines). Representative analyses are shown from pools of cells from 3 mice. Experiments were performed 3 or more times with identical results. **B**) At the indicated days after infection or immunization, splenic cells were stained with anti-CD8, H2K^b^-VNHRFTLV, and the indicated marker-specific antibody labeled prior to analysis by FACS. Numbers in red or green represent mean fluorescence intensity.
(PPT)

Figure S5 **Phenotypic characterization of epitope-specific CD8^+^ T cells induced by infection of genetically deficient C57BL/6 mice with Y strain of** ***T. cruzi*** **.** C57BL/6 mice (WT, MyD88, IL-12/IL-23 KO or IFN-1 receptor KO) were infected s.c. with 10^4^ blood forms of *T. cruzi* Y strain. Control mice were naive mice of the different strains. Fourteen days after infection, the splenic cells of these mice were stained with anti-CD8, H2K^b^-VNHRFTLV, anti-CD95 and anti-CD44 prior to analysis by FACS. The histograms show the expression of the markers: a) H2K^b^-VNHRFTLV and CD8 on splenic cells form infected or control naive CD8^+^ spleen cells; b) CD95 and CD44 on H2K^b^-VNHRFTLV ^+^ (blue lines) or naive CD8^+^ cells (red lines). Numbers in red or blue represent mean fluorescence intensity. Analyses are shown for a representative from 3 mice.
(PPT)

Figure S6 **Phenotypic characterization of specific CD8^+^ T cells of infected and/or AdASP-2 immunized C57BL/6 mice.** A) Mice were immunized i.m. with 2×10^8^ pfu of the indicated recombinant adenovirus and challenged s.c. with 10^4^ trypomastigotes of *T. cruzi*. B) Control cells were CD8^+^ T cells from naive mice (red lines). Splenic cells were stained with anti-CD8, H2K^b^-VNHRFTLV, anti-CD95 and annexin V prior to FACS analysis (blue lines). The histograms show FACS analysis on CD8^+^ cells (Gr. 1) or H2K^b^-VNHRFTLV^+^ CD8^+^ cells (Gr. 2, 3, and 4) stained for CD95 or incorporated BrdU. Representative analyses are shown from pools of cells of 3 mice per experiment. Analyses of each individual mouse provided the same result. The results for the annexin V ligand are expressed as the percentage of positive cells. The experiment was performed 3 times with similar results.
(PPT)

Figure S7 **Specific CD8^+^ T cell-mediated immune responses of infected and/or AdTS immunized BALB/c mice.** **A**) BALB/c mice were immunized i.m. with of Adβ-gal or AdTS vaccine (2×10^8^ pfu/mouse). One week later, half of the mice were challenged s.c. with trypomastigotes of the Brazil strain of *T. cruzi* (10^3^ bloodstream parasites/mouse). **B**) Parasitemia (per mL) was estimated at the indicated days following infection of mice that had been immunized with Adβ-gal (Gr. 2) or AdTS vaccine (Gr. 4). The values of parasitemia were significantly lower in mice from Gr. 4 (*P*<0.05, n = 4). **C**) Twenty eight days after challenge (35 days after adenovirus immunization), we estimated the frequency of splenic H2K^d^-IYNVGQVSI^+^ CD8^+^ cells. The results are presented in terms of each mouse (dots) and medians (bars). **D**) On that day, the splenic cells were cultured in the presence of anti-CD107a and anti-CD28, with or without the peptide IYNVGQVSI. After 12 h, cells were stained for CD8, IFN-γ, and TNF-α. The results are presented as mean ± SD frequencies of splenic CD8^+^ cells of 4 mice. The values of cultures stimulated with peptide IYNVGQVSI were subtracted from those of cultures with medium alone. The asterisks and crosses denote significantly higher numbers of peptide-specific cells than in the group of mice immunized with Adβ-gal or all other groups, respectively (*P*<0.05). **E**) Pie charts show the fraction of peptide-specific cells expressing the indicated molecules. The results are expressed as the mean values for 4 mice per group. The results are representative of 2 independent experiments. The asterisks and crosses denote significantly higher numbers of peptide-specific cells than in the group of mice immunized with Adβ-gal or all other groups, respectively (*P*<0.05).
(PPT)

Figure S8 **Phenotypic characterization of specific CD8^+^ T cells of infected and/or AdTS immunized BALB/c mice.** **A**) Thirty five days after adenovirus immunization, we estimated the frequencies of H2K^d^- IYNVGQVSI^+^ CD8^+^ cells. Because some of these mice were challenged 7 days after immunization, this date represents 28 days after challenge with parasites (Gr. 2 and Gr. 4). FACS charters are from a representative mouse (median) from 3 mice. Numbers represent percentage of splenic cells. **B to D**) Splenic cells were stained for CD8, H2K^d^- IYNVGQVSI, CD95, KLRG1, and CD95L prior to analysis by FACS. The histograms show the expression of the markers on H2K^d^- IYNVGQVSI^+^ CD8^+^ cells (blue lines) or control naive CD8^+^ spleen cells (red lines). Numbers in red or blue represent mean fluorescence intensity. **E**) Kinetics of CD95 expression on H2K^d^- IYNVGQVSI^+^ CD8^+^ cells (blue lines) at different days after inoculation of the parasites. Control cells were from naive mice (red lines). Representative samples (median) are shown from 3 mice per experiment.
(PPT)
