# Supplementary material for: Pathogen-Induced Proapoptotic Phenotype and High CD95 (Fas) Expression Accompany a Suboptimal CD8+ T-Cell Response: Reversal by Adenoviral Vaccine
Source: PLoS Pathog. 2012 May 17;8(5):e1002699. doi: 10.1371/journal.ppat.1002699 (PMC3355083; doi:10.1371/journal.ppat.1002699)
Supplement: Figure S1 — Parasitemia and kinetics of specific CD8+ T cell-mediated immune responses during infection or vaccination. A) Parasitemia of C57BL/6 mice infected s.c. with 104 bloodstream trypomastigotes of T. cruzi B) Mice were infected s.c. or not with T. cruzi trypomastigotes as described above. In parallel, mice were immunized i.m. with AdASP-2 vaccine (2×10^8^ pfu/mouse). At the indicated days, the in vivo cytotoxic activity against target cells coated with peptide VNHRFTLV was determined as described in the Methods Section. The results represent the mean ± SD values for 4 mice per group. The results are representative of 3 independent experiments. C–F) C57BL/6 mice were infected or immunized as described above. Control mice were either naive mice or mice immunized with Adβ-gal (2×10^8^ pfu/mouse). At the indicated days after infection or immunization, these mice had their splenic cells cultured in the presence of anti-CD107a and anti-CD28, with or without the peptide VNHRFTLV. After 12 h, cells were stained for CD8, IFN-γ, and TNF-α. Frequencies were estimated for CD8+ cells expressing the indicated molecules after stimulation in vitro with peptide VNHRFTLV. The results are expressed as the mean ± SD values for 4 mice per group. The values of cultures stimulated with peptide VNHRFTLV were always subtracted from those of cultures with medium alone. Pie charts show the fraction of peptide-specific cells expressing the indicated molecules. The results are expressed as the mean values for 4 mice per group. The asterisks and crosses denote significantly higher numbers of peptide-specific cells than in the group of mice immunized with Adβ-gal or all other groups, respectively (P<0.05). (PPT) [file ppat.1002699.s001.ppt]

## Slide 1
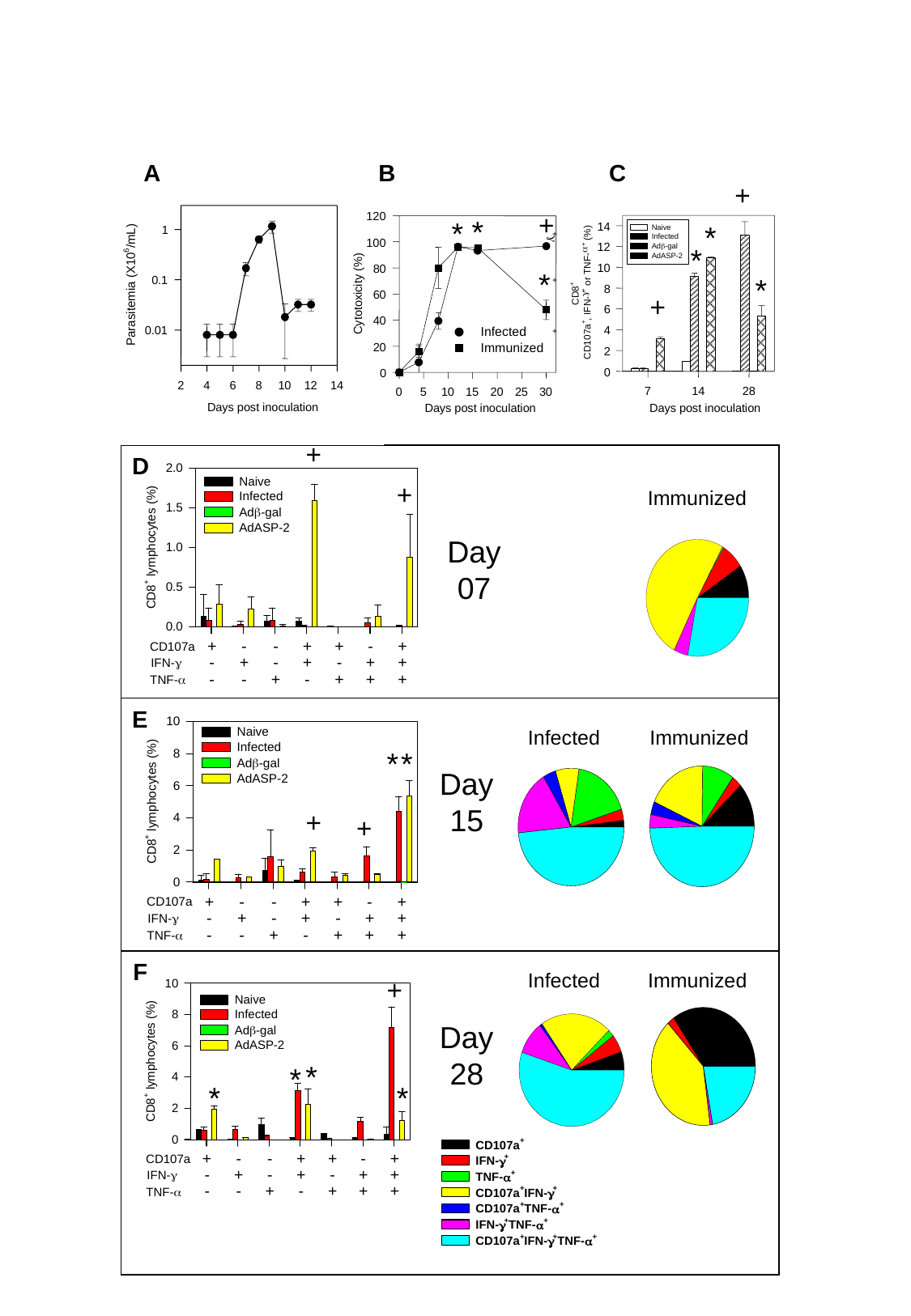

A
A
B
C
+
+
*
14
*
12
10
*
8
+
6
4
2
0
7
14
28
1
0.1
0.01
2
4
6
8
10
12
14
*
+
*
120
+
*
*
*
+
γ
*
100
*
80
*
*
+
+
Cytotoxicity (%)
60
40
+
20
0
0
5
10
15
20
25
30
Days post inoculation
Days post inoculation
Days post inoculation
+
D
+
Immunized
Day
07
E
Infected
Immunized
*
*
Day
15
+
+
F
Infected
Immunized
+
Day
28
*
*
*
*
