# Supplementary figures and images for: Pathogen-Induced Proapoptotic Phenotype and High CD95 (Fas) Expression Accompany a Suboptimal CD8+ T-Cell Response: Reversal by Adenoviral Vaccine
Source: PLoS Pathog. 2012 May 17;8(5):e1002699. doi: 10.1371/journal.ppat.1002699 (PMC3355083; doi:10.1371/journal.ppat.1002699)

## Slide 1
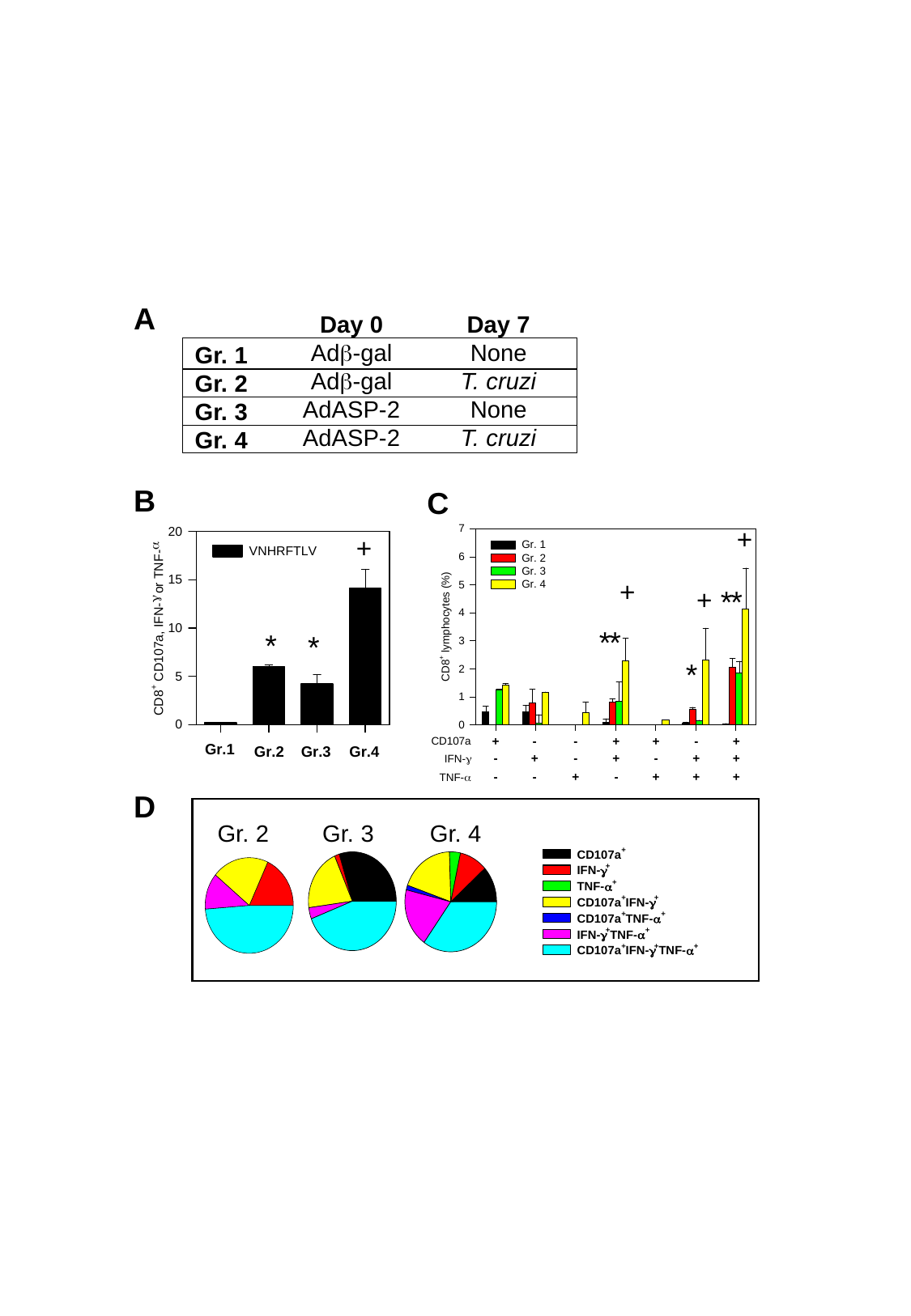

A
Day 0
Adβ-gal
Adβ-gal
AdASP-2
AdASP-2
Day 7
None
T. cruzi
None
T. cruzi
Gr. 1
Gr. 2
Gr. 3
Gr. 4
B
C
+
+
+
+
*
*
*
*
*
*
*
D
Gr. 2
Gr. 3
Gr. 4

Supplement: Figure S2 — Specific CD8+ T cell-mediated immune responses of infected, immunized or infected AdASP-2 immunized C57BL/6 mice. A) C57BL/6 mice were immunized i.m. with 2×10^8^ pfu/mouse of the indicated recombinant adenovirus. Seven days later, half of the mice were challenged s.c. with 104 trypomastigotes of the Y strain of T. cruzi. B) Twenty one, days after immunization with recombinant adenovirus, the splenic cells of these mice were cultured in the presence of anti-CD107a and anti-CD28, with or without the peptide VNHRFTLV. After 12 h, cells were stained with anti-CD8, anti-IFN-γ, and anti-TNF-α. The results are expressed as the total frequency of CD8+ cells stained for any of the indicated molecules (mean ± SD values for 4 mice per group). The values of cultures stimulated with peptide VNHRFTLV were always subtracted from those of cultures with medium alone. C) The same as described above except that the results are expressed as the frequencies of the indicated subpopulation of CD8+ cells stained for CD107a, IFN-γ, and TNF-α (mean ± SD values for 4 mice per group). D) Pie charts show the fraction of peptide-specific cells expressing the indicated molecules. The results are expressed as the mean values for 4 mice per group. The asterisks and crosses denote significantly higher numbers of peptide-specific cells than in the group of mice immunized with Adβ-gal or all other groups, respectively (P<0.05). (PPT) [file ppat.1002699.s002.ppt]

## Slide 1
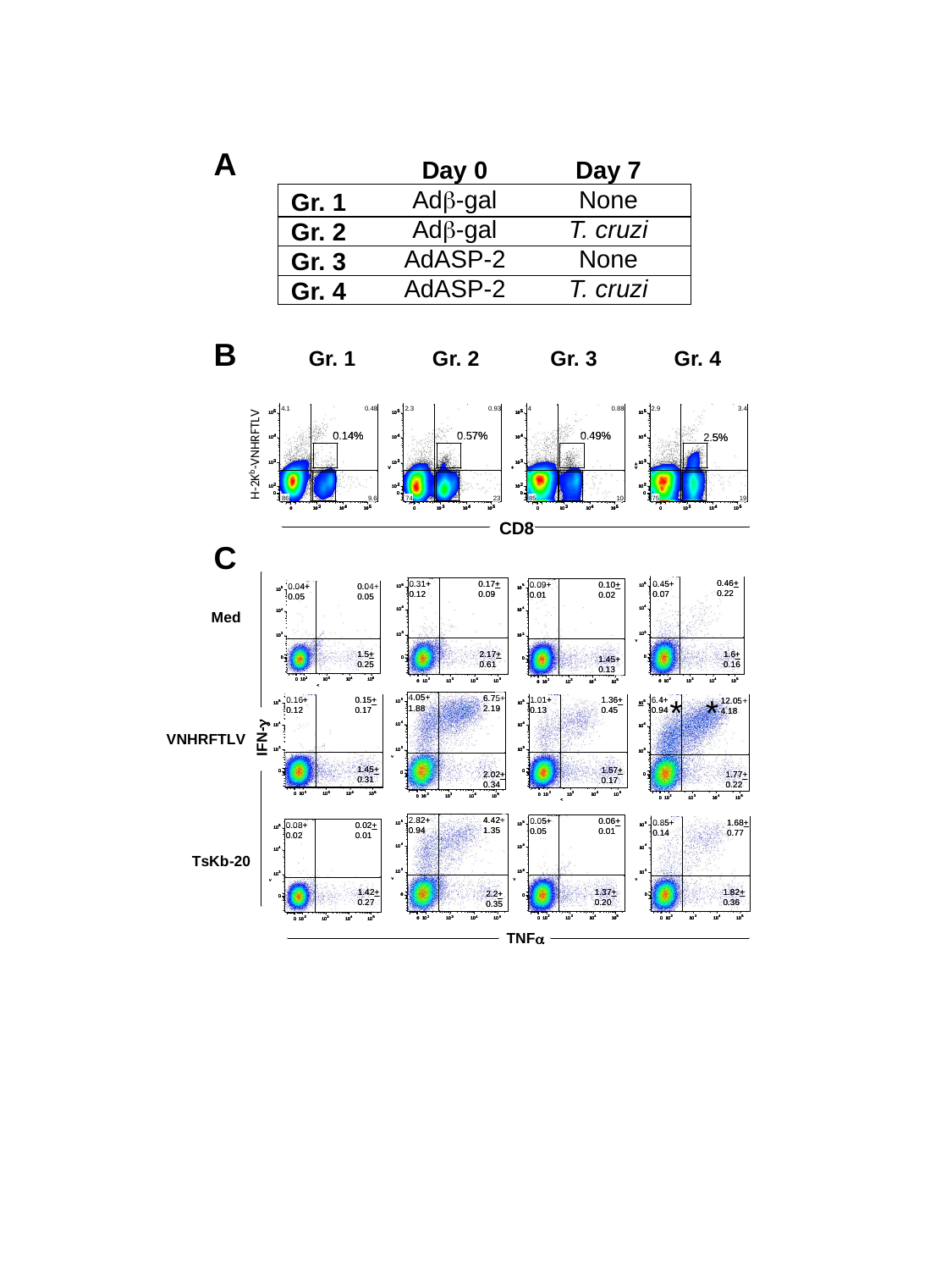

A
Day 0
Adβ-gal
Adβ-gal
AdASP-2
AdASP-2
Day 7
None
T. cruzi
None
T. cruzi
Gr. 1
Gr. 2
Gr. 3
Gr. 4
B
Gr. 1 Gr. 2 Gr. 3 Gr. 4
C

Supplement: Figure S3 — Epitope-specific CD8+ T cell-mediated immune responses of infected and/or immunized immunized C57BL/6 mice. A) C57BL/6 mice were immunized i.m. with 2×10^8^ pfu/mouse of the indicated recombinant adenovirus. Seven days later, half of the mice were challenged s.c. with 104 trypomastigotes of the Y strain of T. cruzi. B) Twenty seven days after immunization with recombinant adenovirus, we estimated the frequency (%) of splenic H2Kb-VNHRFTLV+ CD8+ cells. The results represent a pool of cells from 4 mice per group. C) Twenty seven days after immunization with recombinant adenovirus, the splenic cells of these mice were cultured in the presence of anti-CD28, with or without the peptide VNHRFTLV or TsKb-20. After 12 h, cells were stained with anti-CD8, anti-IFN-γ, and anti-TNF-α. The results are expressed as the total frequency (%) of CD8+ cells stained for any of the indicated molecules (mean ± SD values for 4 mice per group). The asterisks denote significantly higher numbers of peptide-specific cells in Gr. 4 when compared to the same sample of Gr. 2 (P<0.05). (PPT) [file ppat.1002699.s003.ppt]

## Slide 1
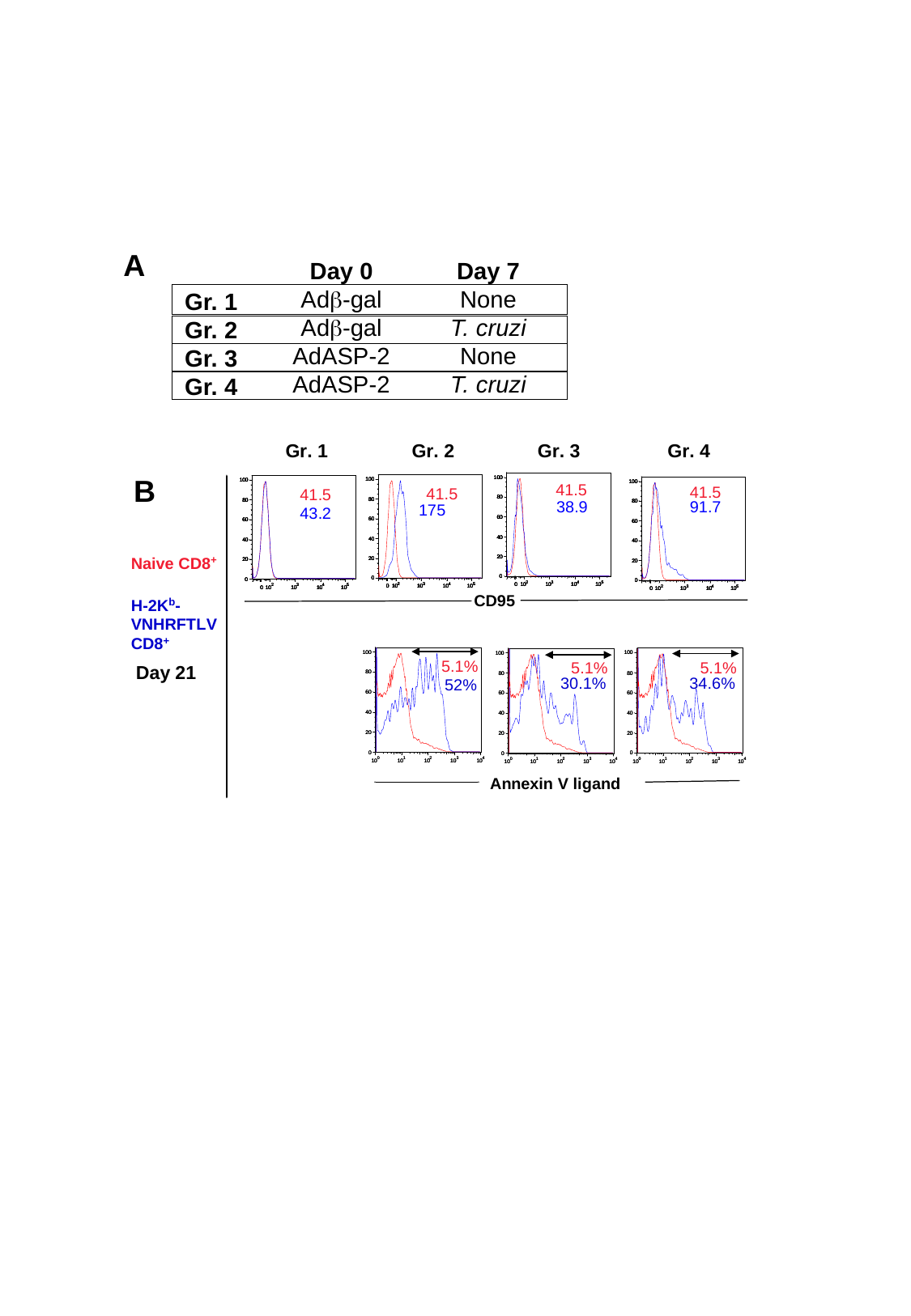

A
Day 0
Adβ-gal
Adβ-gal
AdASP-2
AdASP-2
Day 7
None
T. cruzi
None
T. cruzi
Gr. 1
Gr. 2
Gr. 3
Gr. 4
B

Supplement: Figure S6 — Phenotypic characterization of specific CD8+ T cells of infected and/or AdASP-2 immunized C57BL/6 mice. A) Mice were immunized i.m. with 2×10^8^ pfu of the indicated recombinant adenovirus and challenged s.c. with 104 trypomastigotes of T. cruzi. B) Control cells were CD8+ T cells from naive mice (red lines). Splenic cells were stained with anti-CD8, H2Kb-VNHRFTLV, anti-CD95 and annexin V prior to FACS analysis (blue lines). The histograms show FACS analysis on CD8+ cells (Gr. 1) or H2Kb-VNHRFTLV+ CD8+ cells (Gr. 2, 3, and 4) stained for CD95 or incorporated BrdU. Representative analyses are shown from pools of cells of 3 mice per experiment. Analyses of each individual mouse provided the same result. The results for the annexin V ligand are expressed as the percentage of positive cells. The experiment was performed 3 times with similar results. (PPT) [file ppat.1002699.s006.ppt]

## Slide 1
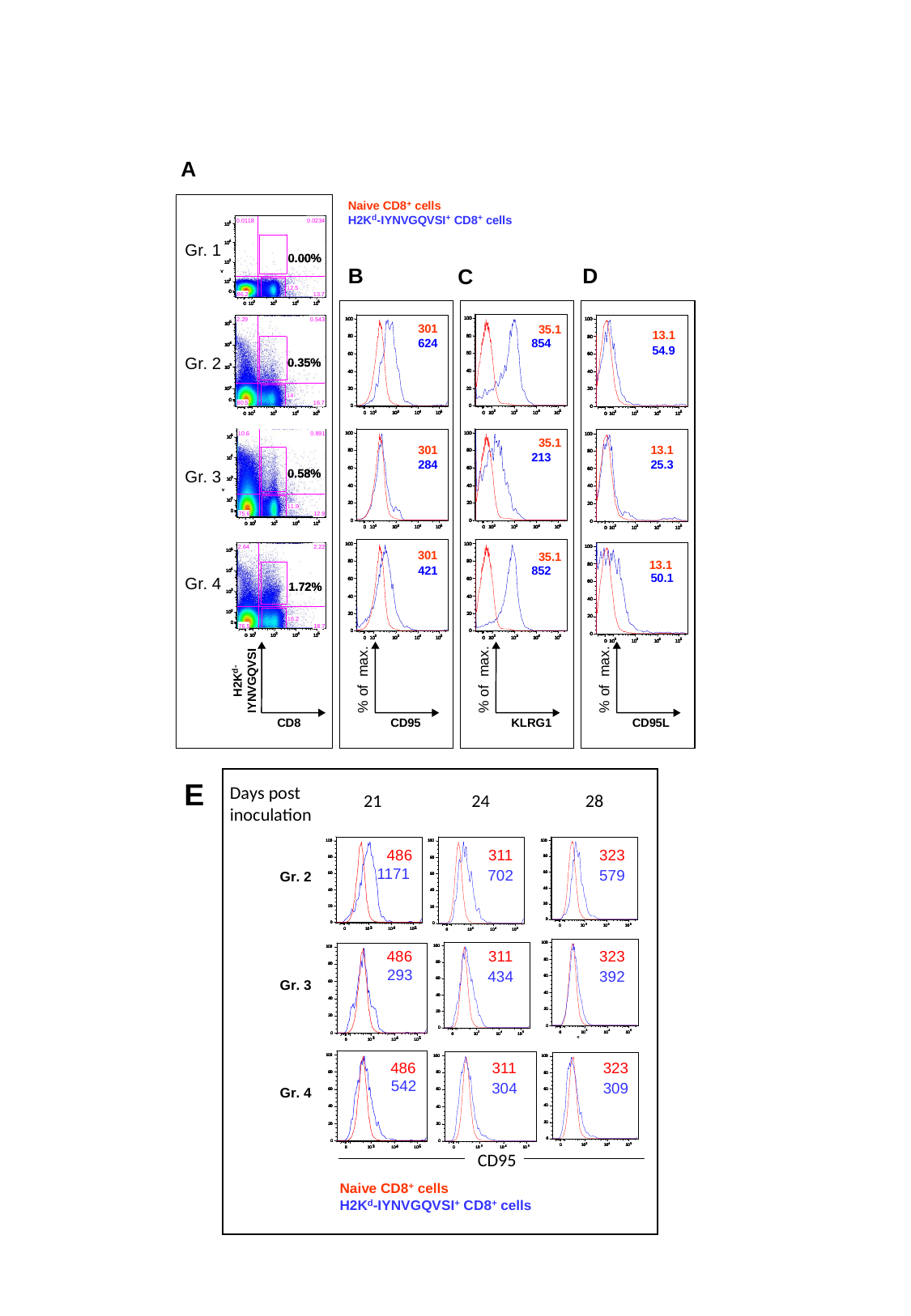

E
486
311
323
1171
702
579
486
311
323
 293
434
392
486
311
323
 542
304
309

Supplement: Figure S8 — Phenotypic characterization of specific CD8+ T cells of infected and/or AdTS immunized BALB/c mice. A) Thirty five days after adenovirus immunization, we estimated the frequencies of H2Kd- IYNVGQVSI+ CD8+ cells. Because some of these mice were challenged 7 days after immunization, this date represents 28 days after challenge with parasites (Gr. 2 and Gr. 4). FACS charters are from a representative mouse (median) from 3 mice. Numbers represent percentage of splenic cells. B to D) Splenic cells were stained for CD8, H2Kd- IYNVGQVSI, CD95, KLRG1, and CD95L prior to analysis by FACS. The histograms show the expression of the markers on H2Kd- IYNVGQVSI+ CD8+ cells (blue lines) or control naive CD8+ spleen cells (red lines). Numbers in red or blue represent mean fluorescence intensity. E) Kinetics of CD95 expression on H2Kd- IYNVGQVSI+ CD8+ cells (blue lines) at different days after inoculation of the parasites. Control cells were from naive mice (red lines). Representative samples (median) are shown from 3 mice per experiment. (PPT) [file ppat.1002699.s008.ppt]
