# Supplementary material for: Pathogen-Induced Proapoptotic Phenotype and High CD95 (Fas) Expression Accompany a Suboptimal CD8+ T-Cell Response: Reversal by Adenoviral Vaccine
Source: PLoS Pathog. 2012 May 17;8(5):e1002699. doi: 10.1371/journal.ppat.1002699 (PMC3355083; doi:10.1371/journal.ppat.1002699)
Supplement: Figure S4 — Phenotypic characterization of specific CD8+ T cells induced by T. cruzi infection or AdASP-2 immunization. C57BL/6 mice were infected or immunized as described in the legend of Figure 2. Control mice were naive mice. A) Twenty-eight or 14 days after infection or immunization, respectively, these mice had their splenic cells stained with anti-CD8, H2Kb-VNHRFTLV, and the indicated marker-specific antibody labeled prior to analysis by FACS. The histograms show the expression of the markers on H2Kb-VNHRFTLV+ CD8+ T cells (green lines) or control naive CD8+ spleen cells (red lines). Representative analyses are shown from pools of cells from 3 mice. Experiments were performed 3 or more times with identical results. B) At the indicated days after infection or immunization, splenic cells were stained with anti-CD8, H2Kb-VNHRFTLV, and the indicated marker-specific antibody labeled prior to analysis by FACS. Numbers in red or green represent mean fluorescence intensity. (PPT) [file ppat.1002699.s004.ppt]

## Slide 1
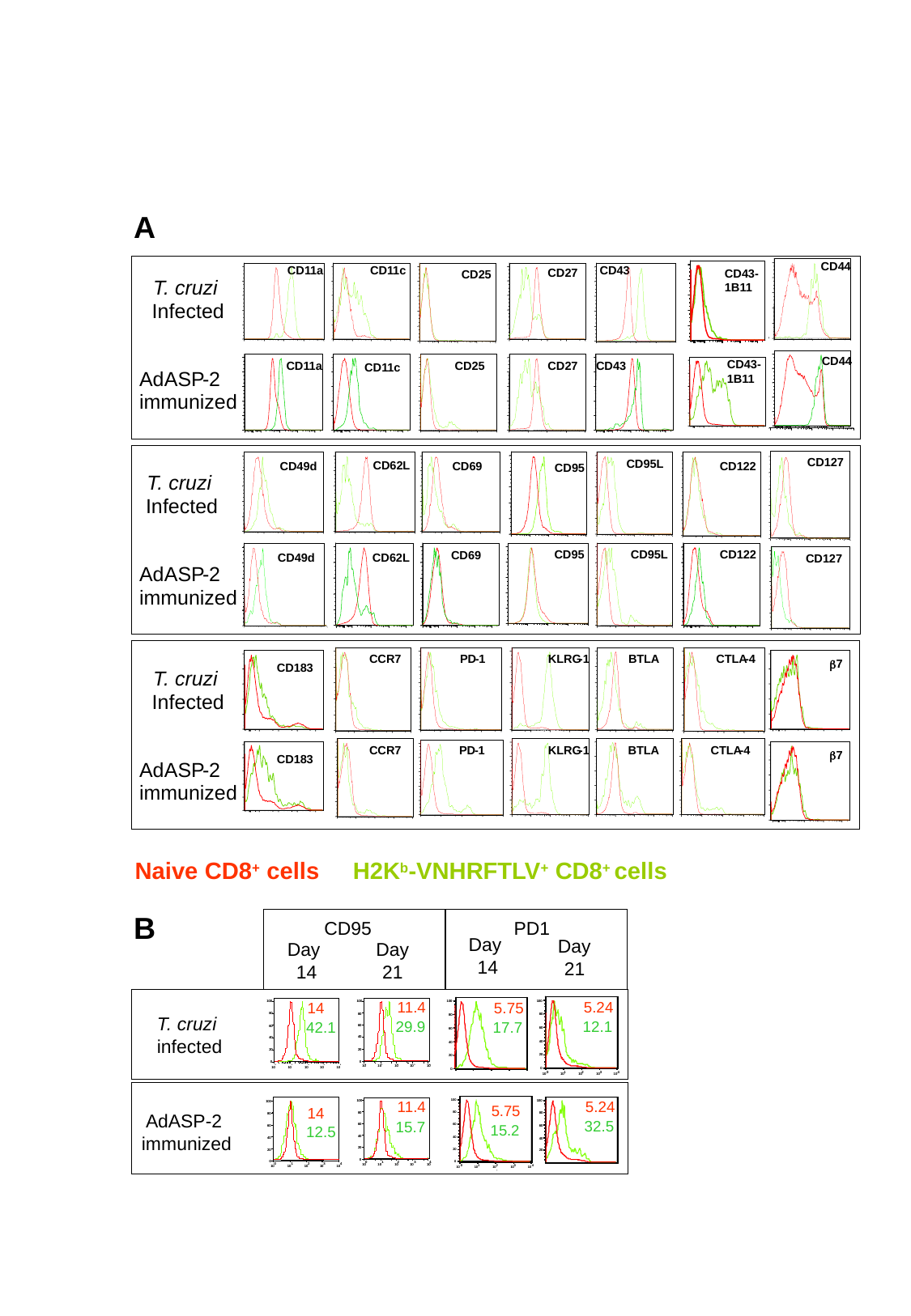

A
CD44
CD44
CD44
CD44
CD11a
CD11a
CD11c
CD11c
CD43
CD43
CD27
CD27
CD43-
1B11
CD25
CD25
T. cruzi
Infected
CD43-
1B11
CD11a
CD11a
CD25
CD27
CD27
CD43
CD43
CD11c
CD11c
AdASP
-
2
immunized
CD127
CD127
CD127
CD127
CD127
CD127
CD127
CD95L
CD95L
CD62L
CD62L
CD49d
CD49d
CD69
CD69
CD122
CD122
CD95
CD95
T. cruzi
Infected
CD95
CD95
CD95L
CD95L
CD122
CD122
CD69
CD69
CD49d
CD49d
CD62L
CD62L
CD49
CD49
CD49
CD49
CD49
AdASP
-
2
immunized
CTLA
CTLA
-
-
4
4
CCR7
CCR7
PD
PD
-
-
1
1
KLRG
KLRG
-
-
1
1
BTLA
BTLA
β7
CD183
T. cruzi
Infected
CCR7
CCR7
CTLA
CTLA
-
-
4
4
BTLA
BTLA
PD
PD
-
-
1
1
KLRG
KLRG
-
-
1
1
β7
CD183
AdASP
-
2
immunized
Naive CD8+ cells H2Kb-VNHRFTLV+ CD8+ cells
B
CD95
PD1
Day
Day
Day
Day
14
21
14
21
5.24
100
100
80
80
12.1
60
60
40
40
20
20
0
0
0
0
1
1
2
2
3
3
4
4
10
10
10
10
10
10
10
10
10
10
5.75
100
100
80
80
17.7
60
60
40
40
20
20
0
0
11.4
100
100
80
80
60
60
40
40
20
20
0
0
10
10
0
0
10
10
1
1
10
10
2
2
10
10
3
3
10
10
4
4
100
100
80
80
60
60
40
40
20
20
0
0
0
0
1
1
2
2
3
3
4
4
10
10
10
10
10
10
10
10
10
10
14
T. cruzi
infected
29.9
42.1
5.24
100
100
80
80
32.5
60
60
40
40
20
20
11.4
100
100
80
80
60
60
40
40
20
20
0
0
0
0
1
1
2
2
3
3
4
4
10
10
10
10
10
10
10
10
10
10
15.7
100
100
80
80
60
60
40
40
20
20
0
0
0
0
1
1
2
2
3
3
4
4
10
10
10
10
10
10
10
10
10
10
14
12.5
AdASP-2
immunized
