# Supplementary material for: Pathogen-Induced Proapoptotic Phenotype and High CD95 (Fas) Expression Accompany a Suboptimal CD8+ T-Cell Response: Reversal by Adenoviral Vaccine
Source: PLoS Pathog. 2012 May 17;8(5):e1002699. doi: 10.1371/journal.ppat.1002699 (PMC3355083; doi:10.1371/journal.ppat.1002699)
Supplement: Figure S5 — Phenotypic characterization of epitope-specific CD8+ T cells induced by infection of genetically deficient C57BL/6 mice with Y strain of T. cruzi . C57BL/6 mice (WT, MyD88, IL-12/IL-23 KO or IFN-1 receptor KO) were infected s.c. with 104 blood forms of T. cruzi Y strain. Control mice were naive mice of the different strains. Fourteen days after infection, the splenic cells of these mice were stained with anti-CD8, H2Kb-VNHRFTLV, anti-CD95 and anti-CD44 prior to analysis by FACS. The histograms show the expression of the markers: a) H2Kb-VNHRFTLV and CD8 on splenic cells form infected or control naive CD8+ spleen cells; b) CD95 and CD44 on H2Kb-VNHRFTLV + (blue lines) or naive CD8+ cells (red lines). Numbers in red or blue represent mean fluorescence intensity. Analyses are shown for a representative from 3 mice. (PPT) [file ppat.1002699.s005.ppt]

## Slide 1
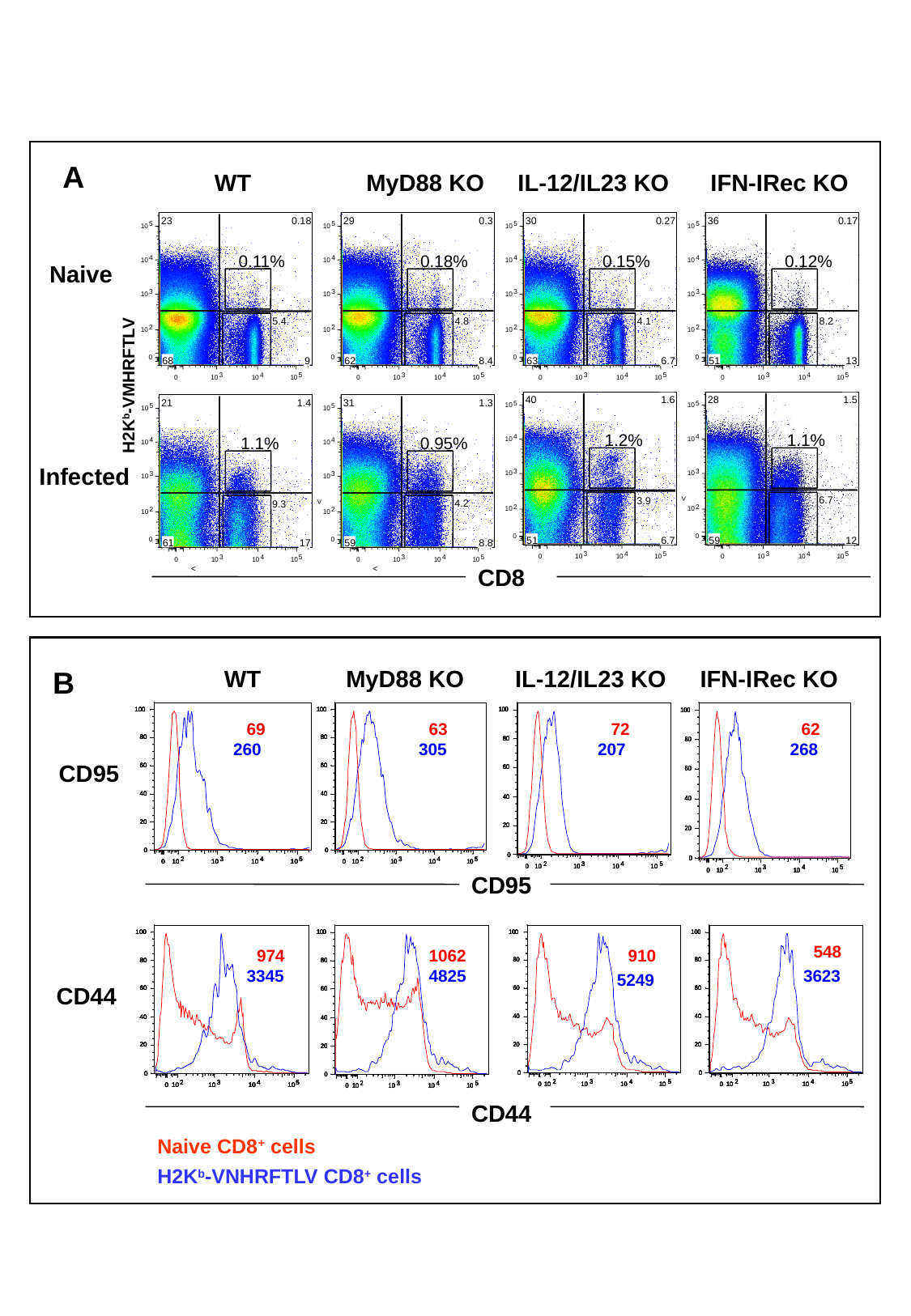

A
WT
MyD88 KO
IL-12/IL23 KO
IFN-IRec KO
5
10
4
10
3
10
2
10
0
3
4
5
0
10
10
10
0.18
23
0.11%
68
9
5.4
5
10
4
10
3
10
2
10
0
3
4
5
0
10
10
10
0.3
29
0.18%
62
8.4
4.8
5
10
4
10
3
10
2
10
0
3
4
5
0
10
10
10
0.27
30
0.15%
63
6.7
4.1
5
10
4
10
3
10
2
10
0
3
4
5
0
10
10
10
0.17
36
0.12%
51
13
8.2
Naive
H2Kb-VMHRFTLV
5
10
4
10
3
10
2
10
0
3
4
5
0
10
10
10
1.6
40
1.2%
51
6.7
3.9
5
10
4
10
3
10
2
10
0
<
3
4
5
0
10
10
10
1.5
28
1.1%
59
12
6.7
5
10
4
10
3
10
2
10
0
3
4
5
0
10
10
10
<
1.4
21
1.1%
61
17
9.3
5
10
4
10
3
10
2
10
0
<
3
4
5
0
10
10
10
<
1.3
31
0.95%
59
8.8
4.2
Infected
CD8
B
WT
MyD88 KO
IL-12/IL23 KO
IFN-IRec KO
69
63
72
62
260
305
207
268
CD95
CD95
548
974
1062
910
3345
4825
3623
5249
CD44
CD44
Naive CD8+ cells
H2Kb-VNHRFTLV CD8+ cells
