# Supplementary material for: Pathogen-Induced Proapoptotic Phenotype and High CD95 (Fas) Expression Accompany a Suboptimal CD8+ T-Cell Response: Reversal by Adenoviral Vaccine
Source: PLoS Pathog. 2012 May 17;8(5):e1002699. doi: 10.1371/journal.ppat.1002699 (PMC3355083; doi:10.1371/journal.ppat.1002699)
Supplement: Figure S7 — Specific CD8+ T cell-mediated immune responses of infected and/or AdTS immunized BALB/c mice. A) BALB/c mice were immunized i.m. with of Adβ-gal or AdTS vaccine (2×10^8^ pfu/mouse). One week later, half of the mice were challenged s.c. with trypomastigotes of the Brazil strain of T. cruzi (103 bloodstream parasites/mouse). B) Parasitemia (per mL) was estimated at the indicated days following infection of mice that had been immunized with Adβ-gal (Gr. 2) or AdTS vaccine (Gr. 4). The values of parasitemia were significantly lower in mice from Gr. 4 (P<0.05, n = 4). C) Twenty eight days after challenge (35 days after adenovirus immunization), we estimated the frequency of splenic H2Kd-IYNVGQVSI+ CD8+ cells. The results are presented in terms of each mouse (dots) and medians (bars). D) On that day, the splenic cells were cultured in the presence of anti-CD107a and anti-CD28, with or without the peptide IYNVGQVSI. After 12 h, cells were stained for CD8, IFN-γ, and TNF-α. The results are presented as mean ± SD frequencies of splenic CD8+ cells of 4 mice. The values of cultures stimulated with peptide IYNVGQVSI were subtracted from those of cultures with medium alone. The asterisks and crosses denote significantly higher numbers of peptide-specific cells than in the group of mice immunized with Adβ-gal or all other groups, respectively (P<0.05). E) Pie charts show the fraction of peptide-specific cells expressing the indicated molecules. The results are expressed as the mean values for 4 mice per group. The results are representative of 2 independent experiments. The asterisks and crosses denote significantly higher numbers of peptide-specific cells than in the group of mice immunized with Adβ-gal or all other groups, respectively (P<0.05). (PPT) [file ppat.1002699.s007.ppt]

## Slide 1
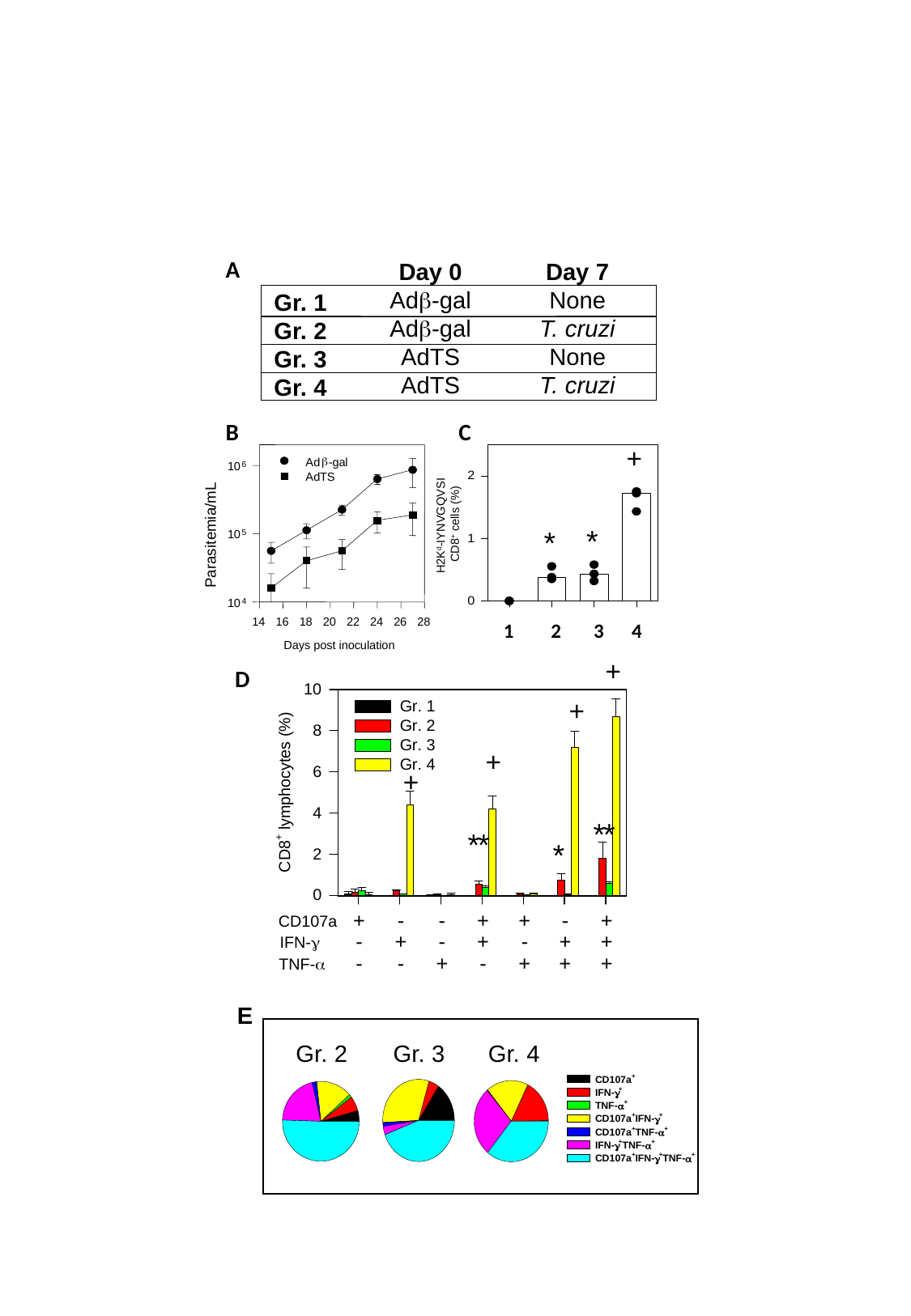

A
Day 7
None
T. cruzi
None
T. cruzi
Gr. 1
Gr. 2
Gr. 3
Gr. 4
Day 0
Adβ-gal
Adβ-gal
AdTS
AdTS
B
C
+
β
Ad
-gal
10
6
AdTS
Parasitemia (X106/mL)
Parasitemia/mL
10
5
10
4
14
16
18
20
22
24
26
28
Days post inoculation
H2Kd-IYNVGQVSI
CD8+ cells (%)
*
*
1 2 3 4
+
D
+
+
+
*
*
*
*
*
E
Gr. 2
Gr. 3
Gr. 4
